# Beyond trophic morphology: stable isotopes reveal ubiquitous versatility in marine turtle trophic ecology

**DOI:** 10.1111/brv.12543

**Published:** 2019-07-24

**Authors:** Christine Figgener, Joseph Bernardo, Pamela T. Plotkin

**Affiliations:** ^1^ Marine Biology Interdisciplinary Program Texas A&M University 3258 TAMU, College Station TX 77843 U.S.A.; ^2^ Department of Biology Texas A&M University 3258 TAMU, College Station TX 77843 U.S.A.; ^3^ Department of Oceanography Texas A&M University 3146 TAMU, College Station TX 77843 U.S.A.; ^4^ Program in Ecology and Evolutionary Biology Texas A&M University 2475 TAMU, College Station TX 77843 U.S.A.; ^5^ Texas Sea Grant, Texas A&M University 4115 TAMU, College Station TX 77843 U.S.A.

**Keywords:** niche variation hypothesis, ecological partitioning, trophic variability, cryptic dietary diversity, marine food webs, ecoinformatics, interspecific competition, intraspecific competition, animal personality, ecological exchangeability

## Abstract

The idea that interspecific variation in trophic morphology among closely related species effectively permits resource partitioning has driven research on ecological radiation since Darwin first described variation in beak morphology among *Geospiza*.

Marine turtles comprise an ecological radiation in which interspecific differences in trophic morphology have similarly been implicated as a pathway to ecopartition the marine realm, in both extant and extinct species. Because marine turtles are charismatic flagship species of conservation concern, their trophic ecology has been studied intensively using stable isotope analyses to gain insights into habitat use and diet, principally to inform conservation management. This legion of studies provides an unparalleled opportunity to examine ecological partitioning across numerous hierarchical levels that heretofore has not been applied to any other ecological radiation. Our contribution aims to provide a quantitative analysis of interspecific variation and a comprehensive review of intraspecific variation in trophic ecology across different hierarchical levels marshalling insights about realised trophic ecology derived from stable isotopes.

We reviewed 113 stable isotope studies, mostly involving single species, and conducted a meta‐analysis of data from adults to elucidate differences in trophic ecology among species. Our study reveals a more intricate hierarchy of ecopartitioning by marine turtles than previously recognised based on trophic morphology and dietary analyses. We found strong statistical support for interspecific partitioning, as well as a continuum of intraspecific trophic sub‐specialisation in most species across several hierarchical levels. This ubiquity of trophic specialisation across many hierarchical levels exposes a far more complex view of trophic ecology and resource‐axis exploitation than suggested by species diversity alone. Not only do species segregate along many widely understood axes such as body size, macrohabitat, and trophic morphology but the general pattern revealed by isotopic studies is one of microhabitat segregation and variation in foraging behaviour within species, within populations, and among individuals.

These findings are highly relevant to conservation management because they imply ecological non‐exchangeability, which introduces a new dimension beyond that of genetic stocks which drives current conservation planning.

Perhaps the most remarkable finding from our data synthesis is that four of six marine turtle species forage across several trophic levels. This pattern is unlike that seen in other large marine predators, which forage at a single trophic level according to stable isotopes. This finding affirms suggestions that marine turtles are robust sentinels of ocean health and likely stabilise marine food webs. This insight has broader significance for studies of marine food webs and trophic ecology of large marine predators.

Beyond insights concerning marine turtle ecology and conservation, our findings also have broader implications for the study of ecological radiations. Particularly, the unrecognised complexity of ecopartitioning beyond that predicted by trophic morphology suggests that this dominant approach in adaptive radiation research likely underestimates the degree of resource overlap and that interspecific disparities in trophic morphology may often over‐predict the degree of realised ecopartitioning. Hence, our findings suggest that stable isotopes can profitably be applied to study other ecological radiations and may reveal trophic variation beyond that reflected by trophic morphology.

## I. INTRODUCTION

**Figure 1:**
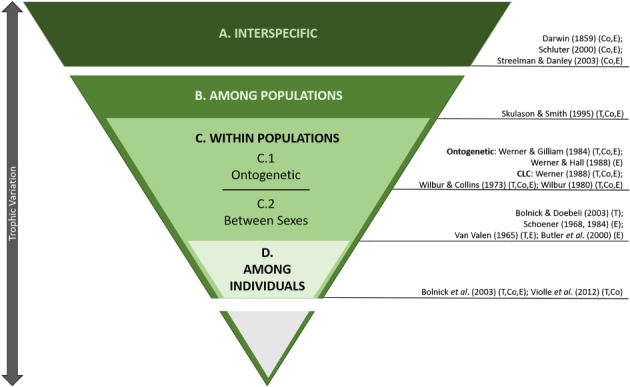
Nested, hierarchical contextualization of trophic variation and studies exemplifying concept in conceptual (Co), theoretical (T), and empirical (E) ways. Trophic variation occurs: (A) among species in adaptive/ecological radiations; (B) among populations, within species; (C) within populations [among different life stages (C.1) and between sexes (C.2)], and (D) among individuals. CLC, complex life cycles.

A key premise of Darwinian evolution is that, because resources are limited, competition is a fundamental driver of evolutionary change. Darwin (1859) argued that interspecific competition causes a ‘struggle for existence’, which ‘will generally be most severe between those forms which are most nearly related to each other in habits, constitution, and structure’ (p. 112). Using this logic, he further hypothesised that resource competition should be more intense within a species than among species. Intraspecific competition occurs among life stages (e.g. between juveniles and adults), between the sexes, and even among individuals within the same life stage and sex. Thus, competition is a continuum encompassing multiple hierarchical levels from interspecific to different levels within species (Fig. 1). Since Darwin's (1859) seminal arguments, ecologists and evolutionary biologists have produced an enormous body of theoretical, conceptual and empirical work that explores how organisms ameliorate both inter‐ and intraspecific competition across all of these hierarchical levels (Fig. 1).

As Darwin (1859) noted, competition is likely most severe among species that are similar in morphology and other attributes, so competition has been studied intensely in adaptive radiations. Adaptive radiation is the process in which organisms diversify rapidly from an ancestral line into a variety of new forms occupying different adaptive zones (Simpson, 1944; Schluter, 2000). A review of vertebrate examples found that radiations unfold through stereotyped stages of diversification, beginning with habitat differentiation and followed by the evolution of divergent, irreversible morphological structures related to divergent trophic ecology (Streelman & Danley, 2003). Because studies of radiations have largely been retrospective, they have typically focused on the terminal and most obvious stage of divergence, morphological divergence, as a proxy to quantify trophic variation among species. Nonetheless, it has been possible to infer the earlier stages by correlating early speciation events with contemporary differences in habitat use.

By contrast, other studies have tried to assess the competitive dynamics of early stages of radiations by examining initial divergence in habitat and morphology and how they relate to trophic ecology using intraspecific systems. Many of the best‐studied examples are from fish that have colonised post‐glacial lakes, which show a consistent signal of foraging habitat segregation (e.g. benthic *versus* pelagic ecomorphs in fish that have colonised post‐glacial lakes (Schluter, 2000) accompanied by morphological manifestations of trophic divergence (Berg *et al.,* 2010; Harrod, Mallela & Kahilainen, 2010; Kahilainen *et al.,* 2004; Knudsen *et al.,* 2006; Muir *et al.,* 2016; Præbel *et al.,* 2013; Schluter, 1993, 1995; Schluter & McPhail, 1992). Even in these examples that examine the putative early stages of radiation, it is extremely difficult to detect a foraging habitat difference without having some signal of morphological differentiation. Notable exceptions come from experimental studies of host‐race formation in insects, in which host‐specialisation evolves without obvious morphological divergence (Feder, Chilcote & Bush, 1988; Feder *et al.,* 2003; Smith & Skúlason, 1996; Via, 1999).

It stands to reason that if foraging habitat diversification is indeed the first stage of radiation, it must be more prevalent than currently recognised because a lack of morphological variation does not necessarily indicate a lack of divergence in habitat use. Most research still mainly relies on morphological differences to recognise that there was an earlier divergence in habitat use. Another reason why divergence in habitat use may also be more common than currently recognised is that detecting divergence in habitat use requires as a first step direct observation of organisms and their pattern of habitat use. This is challenging in species that are difficult to observe, such as those that occupy remote habitats, occur at very low densities, or are highly migratory. We define this undetected habitat divergence that is unaccompanied by a morphological signal as cryptic habitat specialisation.

Although Darwin (1859) recognised that intraspecific competition is likely more severe than interspecific competition, analyses of the mechanisms by which species ameliorate it has lagged far behind analyses of interspecific competition. Ecological niches have typically been characterised at the species level, which implicitly assumes a typological ecology for a given species. However, the niche of a species is the joint response of subpopulations, groups, and individuals to complex ecological and evolutionary processes (Semmens *et al.,* 2009). Thus, the collective differences in niches across relevant levels of hierarchies comprise the niche of a species, known as niche variability (Van Valen, 1965; Semmens *et al.,* 2009). Despite early theoretical (Van Valen, 1965) and empirical (Schoener, 1967, 1968) work aimed at elucidating competitive intraspecific dynamics, detailed consideration of this problem has only emerged in the last three decades. These include analyses of how ontogenetic variation (Werner & Gilliam, 1984), sex‐specific differences (Butler, Schoener & Losos, 2000; Schoener, 1967), and inter‐individual variation (Araujo, Bolnick & Layman, 2011; Bolnick *et al.,* 2007b; Violle *et al.,* 2012) relate to competition (Fig. 1).

The first clear treatment of intraspecific competition was advanced by Van Valen (1965). This idea, now known as the niche‐variation hypothesis (NVH), predicts that populations with wider niches (generalists) are more variable than populations with narrow niches (specialists). As has been the case in analyses of interspecific competition, a search for morphological differences, usually in size, has been the dominant approach in attempts to discover whether individuals within a species partition resources (Schoener, 1967, 1968, 1984; Werner & Gilliam, 1984; Werner & Hall, 1988; Butler, Schoener, & Losos, 2000) according to the NVH. Many studies that have taken this approach have failed to detect evidence of intraspecific resource partitioning (Bolnick *et al.,* 2007b). But again, a morphology‐driven approach is likely to underestimate the extent of ecological partitioning among individuals, because such variation can arise due to behavioural decisions concerning habitat use or prey choice and is not necessarily mediated by morphological phenotypes (Bolnick *et al.,* 2007b). This insight has developed from studies that directly examine dietary variation [gut content analysis (Bolnick *et al.,* 2007b; Costa *et al.,* 2008)]. Both of the latter studies found that more generalised populations exhibit higher among‐individual variation, supporting general predictions of the NVH.

A powerful tool to evaluate the NVH beyond trophic morphology is stable isotope analysis (SIA). Although it has not yet been widely applied to test the NVH *per se*, SIA has provided novel insights on diversification in trophic ecology and habitat use. This approach has confirmed that morphological variation alone may underestimate true levels of trophic diversification. For instance, SIA of aquatic insects, in which there is a strong tradition of assigning species to trophic levels (functional groups) based on their mouthparts, reveals polyphagy across trophic levels not predicted by their trophic morphology (Füreder, Welter & Jackson, 2003; Lancaster *et al.,* 2005; Mihuc & Toetz, 1994; Miyasaka & Genkai‐Kato, 2009).

Stable isotopes are intrinsic markers that are assimilated through the food, water, and gas that enter the body (Rubenstein & Hobson, 2004). The two most commonly used stable isotopes for studies of trophic ecology are stable carbon (^13^C) and stable nitrogen (^15^N). A consumer's stable isotope composition or value is determined by the ratio of light to heavy isotopes (e.g. ^12^C:^13^C or δ^13^C) of its dietary sources (Hobson, 1999). Due to the selectivity of heavier isotopes during metabolic processes, animal tissues tend to be enriched relative to their diet by a discrimination factor of 0–1‰ for δ^13^C (DeNiro & Epstein, 1978) and 3–4‰ for δ^15^N per trophic level (DeNiro & Epstein, 1981), depending on the tissue surveyed. SIA utilises this predictable discrimination from source to consumer to make ecological predictions. In the marine environment, δ^13^C values reflect the value of primary producers in a food chain, which in turn indicates the type of habitat in which an organism is foraging (DeNiro & Epstein, 1978; Hobson, 1999; Rubenstein & Hobson, 2004). Stable nitrogen indicates the trophic position of an organism within its food chain (DeNiro & Epstein, 1981; Hobson, 1999; Rubenstein & Hobson, 2004). Taken together, the combination of δ^13^C and δ^15^N values provides a quantitative isotopic niche, and thus characterises the overall trophic ecology of an individual.

Over the past two decades, SIA has been widely applied to study foraging history and strategies in a wide range of species and biomes. SIAs have been an especially powerful tool to characterise diets and illuminate trophic dynamics in elusive species (e.g. marine or highly migratory). For instance, in marine turtles – the subject of this review – SIA has been used to reconstruct foraging histories of individuals mainly observed in their breeding grounds (Ceriani *et al.,* 2012; Seminoff *et al.,* 2012; Vander Zanden *et al.,* 2015). This body of work has revealed a greater level of complexity in trophic ecology, both among and within species than previously recognised.

In this systematic review, we examine the nature and extent of interspecific and intraspecific variation across all of the hierarchical levels of competition (Fig. 1) using a novel synthesis of stable isotope data for six of the seven extant marine turtle species. Because of conservation and management concerns, a large number of marine turtle populations and management units have been studied using SIA to address a wide variety of questions including foraging patterns and trophic level, habitat use, migration, population connectivity, and physiology at a variety of spatial scales (Figgener, Bernardo & Plotkin, 2019). Unfortunately, little effort has been made to synthesise these findings to address broader evolutionary and ecological questions.

Although these studies were conducted with diverse aims, they provide an opportunity to examine larger‐scale signatures of hierarchical ecological partitioning among marine turtles (Fig. 1). Our review has four main components. We examined interspecific variation in trophic ecology (A in Fig. 1) using a formal meta‐analysis of adult stable isotope values because there were sufficient data. We also synthesised signals of intraspecific variation in trophic ecology across three hierarchical levels (B–D in Fig. 1) using a comparative, descriptive approach, because there were insufficient data to permit a rigorous meta‐analysis at these levels. To our knowledge, no single study across all these hierarchical levels has been conducted previously in any ecological radiation.

## II. BACKGROUND

### (1) Marine turtles as a model system of ecological partitioning

The crown group of marine turtles (Superfamily Chelonioidea) evolved in the mid‐Upper Cretaceous, ∼100–84 million years ago (MYBP) (Gentry, 2017; Pyenson, Kelley & Parham, 2014). A rich fossil record indicates that this radiation comprised up to 27 species that were highly diversified morphologically and ecologically (Cadena & Parham, 2015). The extensive fossil record of marine turtles reveals a large continuum of differentiation along several axes (Parham & Pyenson, 2010; Pyenson, Kelley, & Parham, 2014; Cadena & Parham, 2015). The seven extant species in this monophyletic group reflect this diversity. The Family Cheloniidae is characterised by a keratinised sheath (also called beak or rhamphotheca) covering their jaw bones and a hard shell. It contains six species including the green (*Chelonia mydas* Linnaeus, 1758), loggerhead (*Caretta caretta* Linnaeus, 1758), Kemp's ridley (*Lepidochelys kempii* Garman, 1880), olive ridley (*Lepidochelys olivacea* Eschscholtz, 1829), hawksbill (*Eretmochelys imbricata* Linnaeus, 1766), and flatback turtle (*Natator depressus* Garman, 1880). The Family Dermochelyidae lacks a rhamphotheca and has a leathery shell. It is monotypic, containing the leatherback (*Dermochelys coriacea* Vandelli, 1761) (Table 1).

**Table 1:** Overview of the seven extant marine turtle species: common names, taxonomy, age at sexual maturity (ASM), nesting distribution, trophic ecology and body size. ^1^Spotila (2004); ^2^Bjorndal (1997); ^3^Bolten (2003); ^4^Eckert *et al*. (2012).

					Average adult body size^1^		
Common name	Taxonomy	ASM^1^	Nesting distribution (most northern and southern)	Trophic micro‐habitat^3^/diet^2^	Carapace length [cm]	Mass [kg]	Life‐history^1^: Clutch size # Clutches per season Remigration interval
Loggerhead turtle	Superfamily: Chelonioidea Family: Cheloniidae Genus: *Caretta* ***Caretta caretta*** (Linnaeus, 1758)	17–45	30°N 35°S	benthic/hard‐shelled prey, crustaceans, mollusks	85–124	80–200	97–127 3.9 2–4 years
Green turtle	Superfamily: Chelonioidea Family: Cheloniidae Genus: *Chelonia* ***Chelonia mydas*** (Linnaeus, 1758)	26–44	30°N 23°S	benthic/ seagrass, algae	80–122	65–204	110 3 2.3–5 years
Hawksbill turtle	Superfamily: Chelonioidea Family: Cheloniidae Genus: *Eretmochelys* ***Eretmochelys imbricata*** (Linnaeus, 1843)	17–25	27°N 24°S	benthic/ sponges, soft corals	75–88	43–75	130 3–5 2.9 years
Flatback turtle	Superfamily: Chelonioidea Family: Cheloniidae Genus: *Natator* ***Natator depressus*** (Garman, 1880)	unknown	9°S 24°S	benthic/ echinoderms, shrimps, molluscs, sea pens, bryozoans	75–99	70–90	54 2.8 2.6 years
Kemp's ridley turtle	Superfamily: Chelonioidea Family: Cheloniidae Genus: *Lepidochelys* ***Lepidochelys kempii*** (Garman, 1880)	11–21	35°N 18°N	benthic/ crustaceans	61–76	36–45	110 3 1.5 years
Olive ridley turtle	Superfamily: Chelonioidea Family: Cheloniidae Genus: *Lepidochelys* ***Lepidochelys olivacea*** (Eschscholtz, 1829)	11–16	24°N 30°S	Pelagic–benthic/ crustaceans, molluscs, fish, algae	55–76	36–43	110 2–3 1.7 years

Leatherback turtle	Superfamily: Chelonioidea Family: Dermochelyidae Genus: *Dermochelys* ***Dermochelys coriacea*** (Vandelli, 1761)	12–29^4^	38°N 34°S	pelagic/ soft‐bodied prey: jellyfish, sea salps, tunicates	132–178	250–907	65–85 1–10 2–4 years

Marine turtles are an ideal model group to study ecological partitioning among putative competitors for several reasons. The extant species differ from each other in several ways that are typically associated with ecological radiations including life‐history traits (particularly body size), habitat use, and trophic morphology. Further, the modern species have exhibited morphological stasis over the last 30 million years, suggesting that these differences represent stable ecological strategies.

Thus, like other ecological radiations, the biology of marine turtles simultaneously reflects both signs of resource competition and ecopartitioning. Concerning life histories, marine turtles are similar in some respects (e.g. clutch size, egg size, breeding periodicity), but show striking variation in others, especially body size (Hendrickson, 1980; Van Buskirk & Crowder, 1994; Spotila, 2004). Body‐size divergence among related species is often implicated in the ecological literature as a means of reducing competitive overlap (Smith & Lyons, 2013). Marine turtles span one order of magnitude in adult size from the leatherback turtle (*D. coriacea*), weighing 250–907 kg to the two ridley species (*L. olivacea* and *L. kempii*), weighing 36–43 kg (Spotila, 2004). Within species, the development from hatchling to adult passes through more than two orders of magnitude (Spotila, 2004).

Similarly, concerning habitat use, marine turtles display both spatial overlap and spatial partitioning. On the one hand, marine turtles are highly migratory, travelling thousands of kilometres from feeding grounds to breeding grounds on tropical and subtropical beaches (Plotkin, 2003). These extensive migrations imply broad spatial overlap of ocean habitat. Five of the seven species are widely distributed throughout several ocean basins (Table 1), but species differ in their basin‐wide and within‐basin distributions (Spotila, 2004). The exceptions are *N. depressus*, which is endemic to northern Australasian waters, and *L. kempii*, which mainly inhabits the Gulf of Mexico, but uses other western Atlantic waters. Additionally, most species are confined to tropical and subtropical latitudes for nesting and foraging, except *C. caretta*, which nests and feeds in temperate zones and *D. coriacea*, which nests in the tropics and subtropics but feeds in cold waters at high latitudes. All of these patterns of broad‐scale habitat use imply that marine turtles may often compete for resources. Further, on a finer scale, up to six species may be locally sympatric in regions within ocean basins (see online Supporting information, Table S1). At the finest scale, it is common for several species to overlap in their breeding ranges and on nesting beaches with only weak temporal separation. Often three but up to four species may syntopically use breeding and foraging areas (Cornelius, 1986; Chacon *et al.,* 1996; Chatto & Baker, 2008).

**Figure 2:**
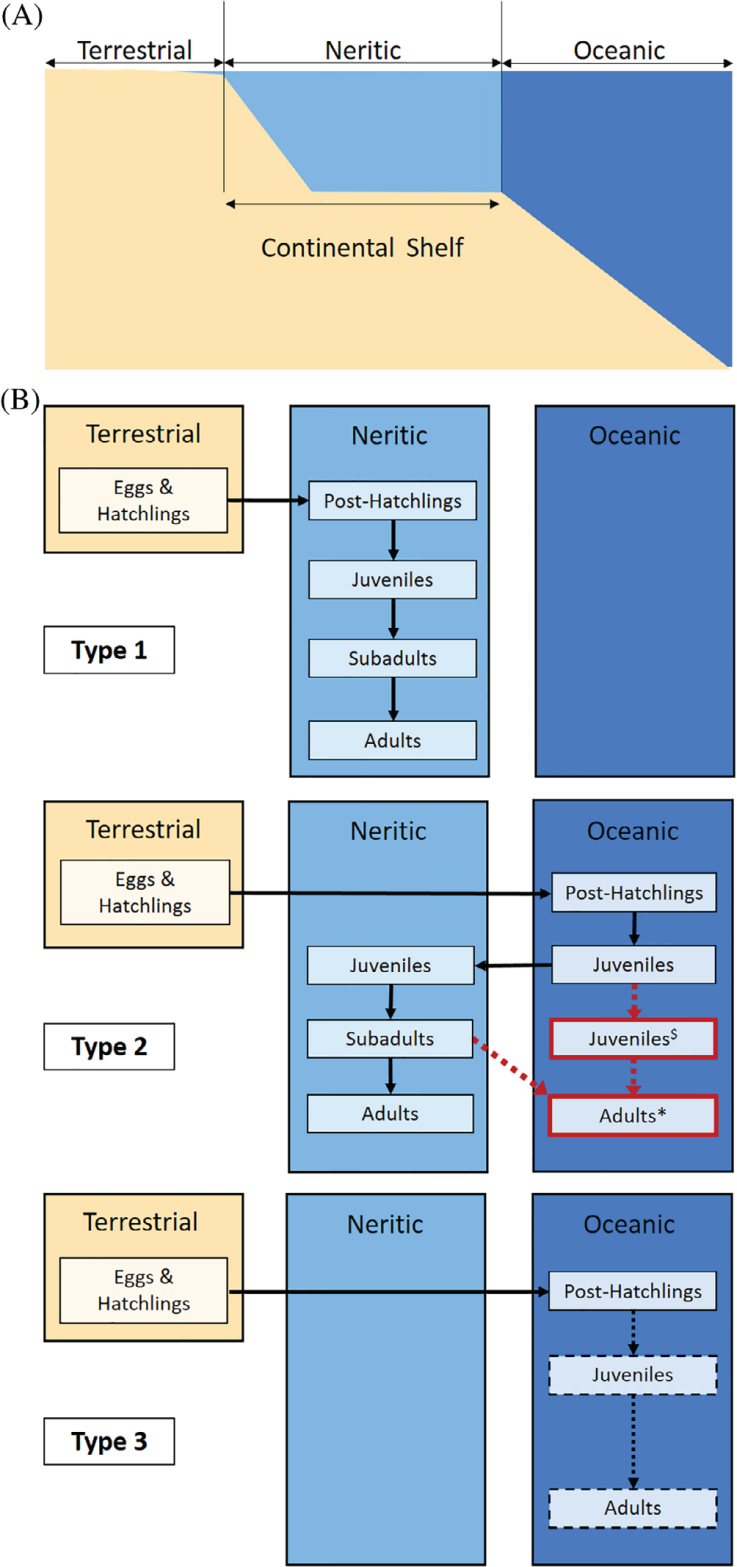
Schematic illustration summarising current knowledge about sea turtle life cycles and their associated marine macrohabitats (modified from Bolten, 2003). (A) Depiction of the three distinct macrohabitats (terrestrial, neritic, oceanic) inhabited by different marine turtle life stages. (B) The three types of life‐history patterns among marine turtle species depicting the sequential use of the three macrohabitats by different developmental stages. In all three panels, solid boxes depict well‐documented associations between life stages and macrohabitats, and solid arrows depict known movements of life stages between macrohabitats. Dashed boxes and arrows depict hypothesised but undocumented associations and movements. The red box and dashed arrows reflect a novel finding of an additional life stage–macrohabitat association of juvenile *C. caretta* (^$^) and adult *C. caretta* and *C. mydas* (*) based on stable isotope analyses (Eder *et al.,* 2012; Hatase *et al.,* 2010, 2013, 2006; McClellan *et al.,* 2010; McClellan & Read, 2007). The Type 1 life cycle is exhibited by *N. depressus*. The Type 2 life cycle is exhibited by *C. caretta*, *C. mydas*, *E. imbricata* and *L. kempii*. The Type 3 life cycle is exhibited by *D. coriacea* and *L. olivacea*.

By contrast, it has long been appreciated that marine turtles exhibit an ecological signature of radiation among habitats (Hendrickson, 1980; Spotila, 2004), at both the ocean basin (macrohabitat) and microhabitat scales (Table 1). At the macrohabitat level, species partition the ocean spatially: horizontally, with regard to the continental shelf (oceanic *versus* neritic, Fig. 2) and vertically, with regard to bathymetry (pelagic, demersal, and benthic, Table 1) (Bjorndal, 1997; Bolten, 2003). *Dermochelys coriacea* and *L. olivacea* principally forage pelagically in oceanic waters. All other species principally forage in neritic waters using one or more layers of the water column. At the microhabitat level, some species are relatively specialised (e.g. seagrass beds, coral reefs) (Table 1) and others are more generalised. These differences in habitat use result in dietary differences, which are reflected in analyses of gut contents (Table 1) (Bjorndal, 1997).

**Figure 3:**
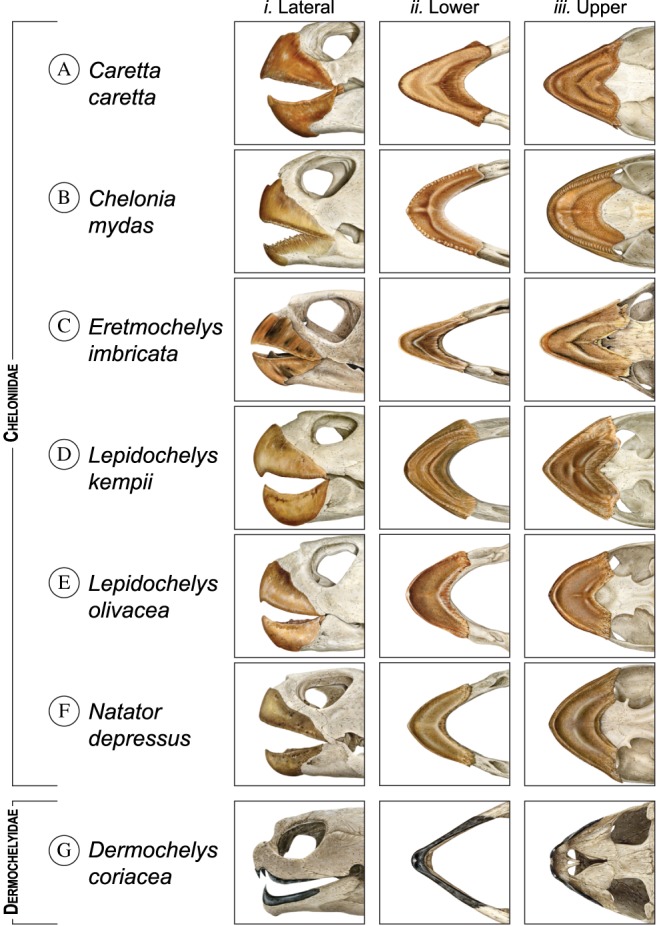
Comparative overview of the trophic morphology of extant marine turtle species. The left panels (*i*) depict lateral views of the skulls; the darker colouration depicts the keratinous sheaths (also called beak or rhamphotheca) that covers the jaws in the six species of Cheloniidae. The single species of Dermochelyidae, *D. coriacea*, lacks a rhamphotheca but possesses skin covering the jaws, which is shown in darker colouration. The middle panels (*ii*) depict dorsal views of the inside of the lower jaw and the right panels (*iii*) depict ventral views of the inside of the upper jaw. These artist's renderings are based on museum specimens housed in the Chelonian Research Institute. High‐resolution versions of these illustrations are provided in Fig. S1. Illustrations by Dawn Witherington.

Finally, concerning trophic morphology, despite the fact that all species have powerful, toothless jaws, the most striking indication of ecopartitioning among marine turtles is the remarkable divergence in the shape of their jaws and beaks (Fig. 3, Fig. S1). The beak, or rhamphotheca, comprises the rhinotheca covering the upper jaw and the gnathotheca covering the lower jaw. The differences in trophic morphology are recognised as feeding ecomorphs based on trophic anatomy and gut‐content analyses. Correlations between trophic morphology and diet in marine turtles have been proffered for both extant (Wyneken, 2003) and fossil (Hirayama, 1994, 1997; Parham & Pyenson, 2010; Gentry, 2017) marine turtles. Interspecific morphological variation in other aspects of head and neck anatomy related to feeding has also been described (Wyneken, 2001, 2003; Jones *et al.,* 2012), further substantiating a link between diet and trophic anatomy.

One ecomorph that has been recognised in extant and fossil species is related to durophagy, which is the reliance upon hard‐shelled prey, such as crustaceans and molluscs. Two extant species are principally durophagous: *C. caretta* has a robust rhamphotheca (Fig. 3A*i*) and wide, crushing surfaces inside the mouth (Fig. 3A*ii*, *iii*). Similarly, the rhamphotheca of *L. kempii* is robust and bears wide ridges for crushing (Fig. 3D*ii*, *iii*). Post‐pelagic individuals feeding in coastal waters have a preponderance of crabs and other crustaceans in their diets, with some molluscs and fish. They also consume algae and seagrasses (Burke, Morreale & Standora, 1994; Burke, Standora & Morreale, 1993; Seney & Musick, 2005; Shaver, 1991).

By contrast, *D. coriacea* (Fig. 3G) is a highly specialised gelativore that feeds on gelatinous prey such as ctenophores, salps (planktonic tunicates) and the planktonic medusae of Cnidaria (Bleakney, 1965; Brongersma, 1969, 1970; Den Hartog, 1979; Den Hartog & Van Nierop, 1984; Duron & Duron, 1980; Duron, Quero & Duron, 1983; Eckert *et al.,* 2012; Paladino & Morreale, 2001), possibly owing to the lack of a keratinized beak. This specialisation is further reflected in its upper jaw (Fig. 3G*i*), which bears two tooth‐like projections used to pierce the air bladders of floating cnidarians (Paladino *et al*., 2001).

Another ecomorph, represented by *E. imbricata* (Fig. 3C), is specialised for spongivory. Its jaws and rhamphotheca, unlike those of all other species, are relatively elongated and narrow (Fig. 3C*ii*, *iii*), terminating in a parrot‐like beak with sharp cutting edges (Fig. 3C*i*). These morphological attributes allow *E. imbricata* to scrape and cut sponges and other reef‐inhabiting anthozoans, such as soft corals and anemones, from hard substrates (Witzell, 1983; Meylan, 1988; Anderes Alvarez & Uchida, 1994; Anderes Alvarez, 2000). It also detaches pieces of corals to access sponges in the interstices of the reef.

The last ecomorph, represented by *C. mydas*, is specialised for herbivory. Its gnathotheca (lower rhamphotheca; Fig. 3B*ii*) bears serrated, sharply ridged edges that occlude against the rhinotheca (upper rhamphotheca; Fig. 3B*iii*), providing the capacity to shear blades of seagrasses, which constitute the majority of their diet (Bjorndal, 1979, 1980, 1985; Forbes, 1993; Mortimer, 1981; Seminoff, Resendiz & Nichols, 2002).

The remaining two species, *L. olivacea and N. depressus*, are omnivorous, exhibiting both durophagous and gelativorous feeding (Montenegro Silva, Bernal Gonzalez & Martinez Guerrero, 1986; Zangerl, Hendrickson & Hendrickson, 1988) and their trophic morphology is so similar that they were once thought to be closely related (Zangerl, Hendrickson, & Hendrickson, 1988).

*Lepidochelys olivacea* is an opportunistic omnivore with a widely varied diet. Studies of stranded turtles report a high degree of durophagy and piscivory (Behera *et al.,* 2015; Colman *et al.,* 2014; Di Beneditto, De Moura & Siciliano, 2015; Spring & Gwyther, 1999; Wildermann & Barrios‐Garrido, 2012). Two studies of freshly killed turtles in Mexico corroborated these findings, but also revealed a high degree of gelativory (mostly salps, but also other tunicates) as well as other soft‐bodied prey including sipunculid worms and bryozoans (Casas‐Andreu & Gómez‐Aguirre, 1980; Montenegro Silva *et al*., 1986). Its trophic morphology reflects this varied diet: *L. olivacea* has the most generalised rhamphotheca in terms of shape and function (Fig. 3E). Within this generalised morphology are several distinct components related to feeding. First, the outer margins of the rhamphotheca bear sharp, cutting edges (Fig. 3E*i*). Second, the gnathotheca bears a sharp, curved ridge along most of its inner margin (Fig. 3E*ii*) which occludes with a similar ridge on the rhinotheca. Third, the rhinotheca bears two elongated, palatal cusps (Fig. 3E*iii*), which are received by two depressions in the gnathotheca (Fig. 3E*ii*) when the beak closes. This functional complex acts like a mortar and pestle to crush and grind hard‐shelled prey. The last distinct feature of the beak is that the rhamphotheca terminates in pointed projections curving toward each other (Fig. 3E*i*). This feature functions similar to a pair of forceps, allowing for fine‐scale picking of small organisms from driftwood (C.F., personal observations) or other substrates. The three‐dimensional relief of the rhamphotheca is not reflected by the underlying, bony elements (Zangerl *et al.,* 1988).

The other omnivore, *N. depressus,* consumes a wide range of gelatinous and other soft‐bodied prey including siphonophores, bryozoans, holothurians, and jellyfish, as well as hard‐shelled prey such as molluscs (Zangerl *et al.,* 1988). This diet diversity is reflected in different components of its trophic anatomy (Zangerl *et al.,* 1988). It has a robust rhamphotheca (Fig. 3F) bearing sharp cutting edges along the outer edge of the jaw (Fig. 3F*i*). Additionally, the rhinotheca bears sharp‐crested ridges along the posterior margin of the secondary palate (Fig. 3F*iii*). Between these cutting surfaces is a flattened, triturating surface (Fig. 3F*iii*). The gnathotheca bears a very prominent, sharp‐edged ridge along the inner margin of the triturating surface, which comes to a sharp, projecting point along the midline (Fig. 3F*ii*). However, unlike in *L. olivacea*, these features are also reflected in the underlying bony architecture of the mandible and the palate.

This summary of ecomorphological differentiation of the seven species of marine turtles and concomitant differentiation of their diets supports the hypothesis that they ecopartition the oceanic realm (Hendrickson, 1980). However, as is found in well‐studied radiations, there also remains some degree of dietary overlap.

### (2) Marine turtle life cycles

An organism's ecology is not defined only by its adult stage, but rather its entire ontogeny (Wiens, 1982; Werner, 1988). Marine turtle population models typically define distinct life stages based on a size‐class system: hatchling, juvenile, subadult, and adult (Fig. 2) (Bolten, 2003; Crouse, Crowder & Caswell, 1987; Heppell, Snover & Crowder, 2003), and thus can be considered to have a complex life cycle, marked by abrupt ontogenetic changes in behaviour and habitat (Werner, 1988). All marine turtles, except *N. depressus* (Fig. 2B, Type 1) (Bolten, 2003), share a general pattern of habitat use among different life stages in which hatchlings migrate from their natal beaches to oceanic nursery habitats (Fig. 2B, Types 2 and 3), where they slowly swim or drift passively within ocean currents (Wyneken & Salmon, 1992; Bjorndal, 1997; Boyle & Limpus, 2008; Mansfield *et al.,* 2014). In the case of *C. caretta*, *C. mydas*, *E. imbricata*, and *L. kempii*, after attaining a threshold size, juveniles enter neritic development habitats (Arthur, 2008; Bjorndal, 1997; Limpus, 1992; Reich, Bjorndal & Bolten, 2007), where they spend most of their lives even after attaining maturity (Fig. 2B, Type 2) (Bjorndal, 1997; Bolten, 2003). By contrast, *D. coriacea* and *L. olivacea* adults range in the open ocean between nesting seasons and little is known about juvenile and subadult stages after the initial oceanic stages, but they are thought to remain oceanic (Fig. 2B, Type 3) (Bjorndal, 1997; Plotkin, 2010; Avens *et al.,* 2013). After reaching maturity, adults of each species migrate at intervals between foraging grounds and distant nesting sites, with females exhibiting high site fidelity over many years (Limpus, 1992; Balazs, 1994; Miller, 1997; Plotkin, 2003). Males also exhibit site fidelity, returning to the same breeding areas (waters adjacent to nesting beaches) annually for mating (Hays *et al.,* 2010; James, Eckert & Myers, 2005a; Plotkin, 2003; Plotkin *et al.,* 1996).

## III. METHODS

### (1) Literature review

We conducted a systematic review of 130 studies analysing stable isotopes in marine turtle tissues and summarised those (*N* = 113) that are primarily concerned with the foraging ecology of marine turtles using δ^13^C and δ^15^N values. Our aim was to analyse interspecific differences and highlight examples of intraspecific and intrapopulation variation in isotopic niche and its possible effects on the mitigation of competition at different hierarchical levels (Fig. 1). Tables S2 and S3 summarise the distribution of studies among species, basins, and broader study topics. A detailed description of the selection and review process, as well as a summary of the data set, is available in Figgener, Bernardo, & Plotkin (2019). The full data set is available as MarTurtSI database on *Dryad* (https://doi.org/10.5061/dryad.3v060tq).

### (2) Meta‐analysis

In addition to the literature review, we used this novel data synthesis to conduct a meta‐analysis of stable isotope composition of adults across six species to seek emergent patterns by comparing among‐species differences, and place them into the general context of marine turtle ecology and evolution. We assessed whether interspecific variation in trophic niche suggested by previous studies is reflected in isotopic values. We confined our analysis to adults for two reasons. First, there is substantial and complex ontogenetic variation in isotopic values among immature life‐history stages (see Section IV.3*a*). Second, growth rate has been shown to affect the fractionation and resulting tissue isotope values (Reich, Bjorndal & del Rio, 2008; Vander Zanden *et al.,* 2012), but because growth slows significantly after turtles attain sexual maturity, comparisons among adults are more straightforward (Chaloupka & Limpus, 1997; Limpus & Chaloupka, 1997).

We obtained mean values of δ^13^C and δ^15^N estimated in adult individuals from published studies of six species (there are no studies of *N. depressus*) (Table 2). We accepted means from studies of any tissue that reported the origin of samples, sample size, and either standard deviation or standard error within one nesting population or foraging area. Alternatively, we also accepted values from studies for which we could compute means and standard errors from either full supplementary data sets if available, or from published graphs from which we extracted raw data using PlotDigitizer 2.6.8 (Huwaldt, 2001). In cases where multiple estimates existed for the same species or populations from different studies, workers, or localities, we accepted all estimates. The full data set used in this meta‐analysis is available in *Dryad* as part of the MarTurtSI database (Figgener *et al.,* 2019).

**Table 2:** Summary statistics of δ^13^C and δ^15^N values from 91 data points for adult marine turtles used in our meta‐analysis

			δ^13^C values			δ^15^N values		
				Range			Range	
	Species	*N*	CV	Minimum	Maximum	CV	Minimum	Maximum
cheloniidae	*Caretta caretta*	48	−0.0932	−18.9	−11.4	0.209	7.3	16.6
	*Chelonia mydas*	9	−0.300	−17.4	− 7.6	0.204	5.1	9.2
	*Eretmochelys imbricata*	4	−0.092	−17.9	−14.4	0.226	5.9	10.5
	*Lepidochelys kempii*	1	NA	−17.9	−17.9	NA	11.2	11.2
	*Lepidochelys olivacea*	7	−0.074	−18.4	−15.5	0.157	9.7	14.3

dermochelyidae	*Dermochelys coriacea*	22	−0.058	−21.1	−16.4	0.135	9.5	16.2

CV, coefficient of variation.

Fifty studies yielded 91 mean stable isotope values that met the minimum selection criteria for inclusion in the meta‐analysis (Figgener *et al.,* 2019) (doi: https://doi.org/10.5061/dryad.3v060tq). The resulting data set was unbalanced in several ways. First, there is a great deal of variation in the number of observations for each species, with *C. caretta* yielding most data points (*N* = 48), only one study of *L. kempii*, and none for *N. depressus* (Table 2). Second, not all ocean basins were surveyed with the same effort (more studies in the Atlantic than any other ocean basin). Additionally, when comparing different ocean basins, stable isotope composition might vary independently of actual differences in foraging strategies, because basins differ in their nutrient cycles and oceanographic features (McMahon, Hamady & Thorrold, 2013). Third, there was great heterogeneity in which tissues were sampled across species, with skin being the most common. For example, one species might have been studied using one tissue in one basin and a different tissue in another (see also Pearson *et al.,* 2017). Comparing stable isotope values estimated from different tissues could be problematic because they have different discrimination factors depending on inherent synthetic pathways (Biasatti, 2004; Reich, Bjorndal, & del Rio, 2008; Seminoff *et al.,* 2009, 2006), and also reflect different times in the foraging history of an individual (Rosenblatt & Heithaus, 2012). Further, we lack a comprehensive framework to compare stable isotope values across all combinations of sampled tissues and across all species. Although a few studies have proposed conversion factors for some pairs of tissues, they were typically within a single species and life stage (Ceriani *et al.,* 2014; Kaufman *et al.,* 2014; Tomaszewicz *et al.,* 2017a). Hence, there is no common currency that would permit standardised comparison across all tissues. As a result, the imbalance of the data and the noise introduced by comparing different ocean basins and different tissues dictated the type of analyses we were able to conduct.

**Figure 4:**
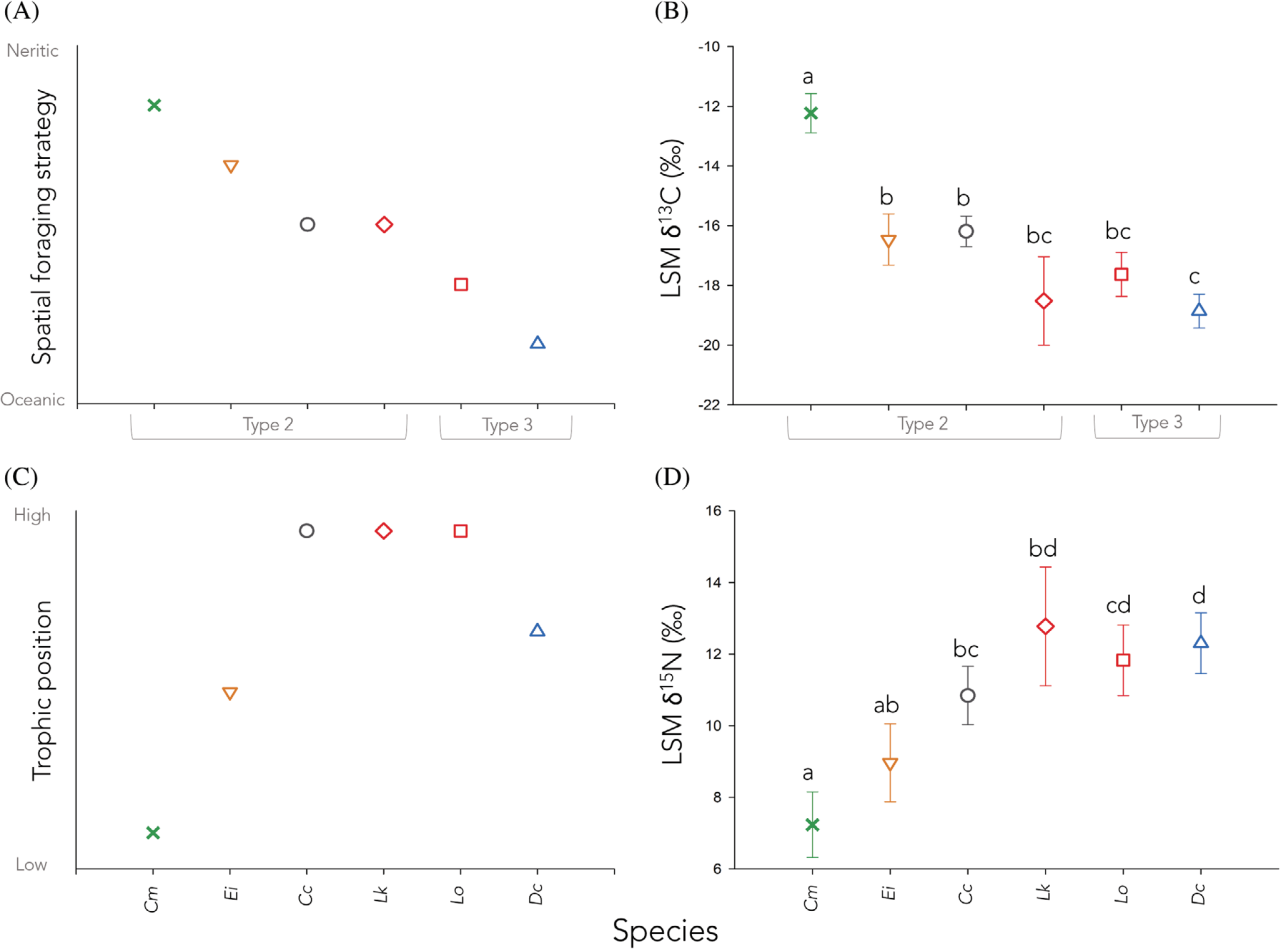
Summary of predicted and observed spatial foraging strategies (δ^13^C, A, B) and trophic position (δ^15^N, C, D) of adults of six marine turtle species (*Cc*, *C. caretta*; *Cm*, *C. mydas*; *Dc*, *D. coriacea*; *Ei*, *E. imbricata*; *Lk*, *L. kempii*; *Lo*, *L. olivacea*). A and C show our predictions (see Section IV.1), and B and D show the species' least‐square means (LSMs) from the linear mixed‐effect models (see Table 3). Statistically significant differences among species determined using Tukey honest significant difference (THSD) *post hoc* tests are indicated by different letters; species that share a letter are not significantly different. In A and B the life‐cycle macrohabitat type (see Fig. 2) is indicated for each species.

We used current understanding of marine turtle foraging ecology and stable isotope gradients in the marine realm (Rubenstein & Hobson, 2004) to generate two *a priori* predictions (Fig. 4A, C) of the rank order among species for δ^13^C and δ^15^N values. The first prediction (Fig. 4A) concerns expected spatial foraging strategies (reflected by δ^13^C) as suggested by studies of spatial macrohabitat use (Fig. 2) (Bolten, 2003; Plotkin, 2003) and microhabitat use (Table 1) (Bjorndal, 1997). The second prediction (Fig. 4C) concerns the expected trophic level of each species (reflected by δ^15^N) based on general diets as suggested by studies of gut contents and known prey species (Table 1) (Bjorndal, 1997), as well as a previous study that determined the trophic level of juvenile and adult *C. caretta*, *C. mydas*, and *D. coriacea* in three sampling locations using stable isotopes (Godley *et al.,* 1998)

**Figure 5:**
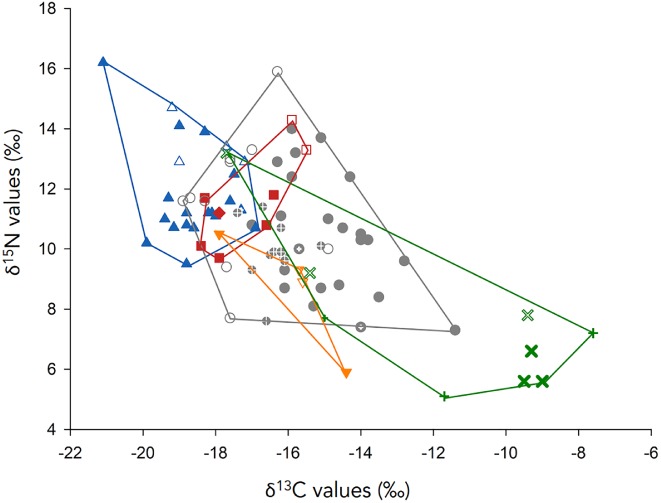
Scatterplot of 91 means from estimates of δ^13^C and δ^15^N in adults of six marine turtle species (*C. caretta*, dark grey circle; *C. mydas*, green cross; *D. coriacea*, blue triangle; *E. imbricata*, orange inverted triangle; *L. kempii*, red diamond, *L. olivacea*, red square) within four ocean basins (Atlantic ocean, filled symbols; Mediterranean sea, large plus signs within symbols; Indian ocean, small plus signs within symbols; Pacific ocean, open symbols) Each point represents a single population. Data are summarised in Figgener *et al*. (2019) and raw data can be found in *Dryad* (https://doi.org/10.5061/dryad.3v060tq). A maximum convex hull is drawn around all points for a given species to facilitate visual comparison.

To gain an overview of species differences as well as intraspecific variation in the data, we first plotted all δ^13^C values *versus* δ^15^N values (Fig. 5). Further, to understand inter‐ and intraspecific variation due to tissue and basin, we conducted exploratory data analyses by first comparing species‐specific isotope values obtained from different tissues but within a single basin (Atlantic, the basin with most estimates) and second comparing species‐specific isotope values obtained from different basins but within the same tissue (skin, the tissue with most estimates). In these analyses we computed separate nested analyses of variance (ANOVAs) of the ratios of each isotope (^13^C, ^15^N) within a single basin and tissue, respectively (Table S4, Table S5, Fig. S2). We took among‐tissue and among‐basin effects into account in our subsequent hypothesis‐testing model.

We evaluated our *a priori* hypotheses concerning interspecific differences in stable isotope composition reflecting spatial foraging strategy (δ^13^C) and trophic level (δ^15^N), and their rank order among species in three steps.

First, to evaluate whether there are interspecific differences, we fitted two separate linear mixed‐effect models for each isotope using the *lme4* package (Bates *et al.,* 2015) in R (R Core Team, 2018). The first model contained species as a fixed factor and tissue (1|Tissue), basin (1|Basin), and an interaction term between tissue and basin (1|Tissue:Basin) as random, blocking factors to account for the heterogeneity and unbalance of the data described above. The second model only included the random, blocking factors. To test for the overall effect of species, we then compared the two models using the Akaike Information Criterion (AIC) and performed a conditional *F*‐test using the Kenward–Roger approximation (Luke, 2017) with the *pbkrtest* package in R (Halekoh & Højsgaard, 2014) To test for pairwise species' differences, we computed Tukey Honestly Significant Difference tests (THSDs) of the resulting least‐squares means between species using the *multcomp* package (Hothorn, Bretz & Westfall, 2008).

Second, to compare the rank order of species against our *a priori* predictions we used Spearman rank correlation on all estimates and separately on the least‐squares means from the linear mixed‐effect models. The Spearman correlation coefficient (*ρ)* ranges from +1 (perfect association) to −1 (inverse association); a *ρ* of zero indicates no association between ranks.

Lastly, to evaluate intraspecific differences we calculated the coefficient of variation for each species (Table 2, Table S6).

## IV. RESULTS

### (1) Variation in trophic ecology among species – a meta‐analysis

Our meta‐analyses of stable isotope composition across six species and multiple ocean basins is the first comprehensive synthesis that permits objective evaluation of the long‐standing hypothesis that marine turtle species effectively ecopartition the marine realm (A in Fig. 1).

Our comparison of the paired mixed‐effect models (conducted for δ^13^C and δ^15^N separately) testing for species differences indicated that the models including species performed far better than the models that did not include species (Table 3): the effect of species was highly significant for both ^13^C (*F*(5) = 25.438, *P*(>*F*) = 6.451e^−15^) and ^15^N (*F*(5) = 9.7253, *P*(>*F*) = 3.628e^−07^) (Table 3). The total random variation not explained by species is 2.8% for δ^13^C, and 5.4% for δ^15^N. Of the random variation in δ^13^C not explained by species only about 2% was due to the interaction of tissue and basin, 28% was due to tissue, 10% was due to basin, and the remaining 60% was unexplained by either factor. Of the random variation in δ^15^N not explained by species only 11% was due to the interaction of tissue and basin, 37% was due to tissue, 18% was due to basin, and the remaining 34% was unexplained by either factor.

**Table 3:** Summary of two linear mixed‐effect models that were used to test for the effect of species in explaining the variation in values among marine turtle species within each of two isotopes (^13^C and ^15^N). Species was treated as fixed factor, and tissue and basin as random blocking factors. In addition, a random term for the interaction between tissue and basin was included

	#	Model	AIC	Marginal *R* ^2^	Conditional *R* ^2^
**δ** ^**13**^ **C**	**1**	δ^13^C ∼ Species + (1|Tissue) + (1|Basin) (1|Tissue:Basin)	327.131	0.553	0.729
	**2**	δ^13^C ∼ (1|Tissue) + (1|Basin) (1|Tissue:Basin)	410.378	NA	NA

**δ** ^**15**^ **N**	**1**	δ^15^N ∼ Species + (1|Tissue) + (1|Basin) (1|Tissue:Basin)	346.045	0.285	0.756
	**2**	δ^15^N ∼ (1|Tissue) + (1|Basin) (1|Tissue:Basin)	397.200	NA	NA

AIC, Akaike Information Criterion.

The THSD *post hoc* tests revealed three distinct spatial foraging strategies (δ^13^C) and two distinct clusters of trophic levels (δ^15^N) among the six species for which data were available. With respect to spatial foraging strategy (Fig. 4B), *C. mydas* (group a) was distinct from all other species; *E. imbricata* and *C. caretta* comprised a second group (group b) and *D. coriacea* a third (group c). Both species of *Lepidochelys* were intermediate and not significantly different from groups b or c. The Spearman rank‐order correlation between our *a priori* species ranks of δ^13^C values (Fig. 4A) and both all estimates and the least‐squares mean species ranks was significant (*ρ*_ALL_(4) = 0.81, *P* = 0.05; *ρ*_LSQM_(4) = 0.81, *P* = 0.05). With respect to δ^15^N values (Fig. 4D), while two significantly different groups were identified (a and d), the differences were not as distinct for δ^15^N as they were for δ^13^C, owing to larger intraspecific variance than in δ^13^C values. Nonetheless, the Spearman correlation of our *a priori* predictions of δ^15^N was significant (*ρ*_ALL_(4) = 0.89; *P* = 0.025; *ρ*_LSQM_(4) = 0.83; *P* = 0.025).

The congruence between the rank orders of our *a priori* predictions based on spatial foraging strategy, gut content analyses, and trophic morphology, and the rank order of the stable isotope estimates broadly corroborates the hypothesis of ecopartitioning among marine turtle species (Fig. 4). However, far more complexity and overlap among species are revealed by the stable isotope data (Fig. 5, Fig. S2).

Spatial patterns in foraging for adults as predicted by the patterns in Fig. 2 are expected to correlate with δ^13^C values because oceanic primary producers (planktonic macroalgae and marine phytoplankton) primarily use the C_3_ photosynthetic pathway (Fry, 1996), whereas terrestrial plants, the source of most nearshore carbon, use both C_3_ and C_4_/crassulacean acid metabolism (CAM) photosynthetic pathways. This results in very distinct signatures for oceanic and near‐shore habitats (Rubenstein & Hobson, 2004). Additionally, the δ^13^C values of seagrasses (e.g. genera *Zosta* and *Halophila*), a principal component of the diet of *C. mydas*, resemble those of terrestrial C_4_ plants (Andrews & Abel, 1979; Beer, Shomer‐Ilan & Waisel, 1980; Hemminga & Mateo, 1996).

While the pattern we observed in δ^13^C was congruent with our predictions based on assignment of species according to macrohabitat (neritic *versus* oceanic) and microhabitat (benthic, pelagic etc.) use, the three significantly distinct groups (Fig. 4B) did not perfectly coincide with adult spatial life‐cycle patterns (Fig. 2). Group a comprised a single species, the coastally foraging and largely herbivorous *C. mydas* which was distinct from all other species including others sharing the Type 2 life‐cycle pattern (*C. caretta*, *E. imbricata*, and *L. kempii*; Fig. 2). On the opposite extreme, group c included the two highly oceanic species sharing the Type 3 life‐cycle pattern (*D. coriacea*, *L. olivacea*), but it also included one species with the Type 2 life‐cycle pattern (*L. kempii*). A third group (b) was intermediate and contained mainly Type 2 species, with the addition of *L. olivacea*. It is noteworthy that the two *Lepidochelys* species are more similar to each other than to the other species in their respective life‐cycle pattern groups.

This imperfect congruence between life‐cycle patterns and species average δ^13^C values indicates far greater complexity in spatial foraging strategies within and among marine turtle species. Indeed, when the intraspecific and interspecific variation in δ^13^C values is viewed simultaneously, the spatial foraging strategies of marine turtles are clearly seen as a continuum (Fig. 5). Hence, the general life‐cycle pattern classification (Fig. 2) obscures fine‐scale differences in spatial habitat use among and within species, even within the same macrohabitat foraging group.

In contrast to the congruence of spatial habitat use and δ^13^C values, trophic level, estimated by δ^15^N, is not likely to be predicted cleanly from trophic morphology. This is because, within a given trophic morphology, species are expected to feed across trophic levels (Bjorndal, 1997). For instance*, D. coriacea*, a specialized gelativore, feeds on both primary consumers such as filter‐feeding tunicates, but also on carnivorous Cnidarians such as Portuguese man ‘o war (*Physalia physalis*) and lion's mane (*Cyanea capillata*), both of which are known to feed on fish and which are thus at least tertiary consumers (Paladino & Morreale, 2001). In the case of *C. caretta*, a specialised durophage, stomach content analyses indicate that it feeds on both low‐trophic‐level, filter‐feeding molluscs, and high‐trophic‐level, carnivorous crustaceans (Plotkin, Wicksten & Amos, 1993). By contrast, *C. mydas*, whose trophic morphology is specialised for herbivory, is expected to forage only as a primary consumer.

Our analyses revealed four foraging groups (a–d) that overlapped among species (Fig. 4D). Group a contains *Chelonia mydas* and *E. imbricata*; group b contains *E. imbricata*, *C. caretta*, and *L. kempii*; group c contains *C. caretta* and *L. olivacea*; and group d contains *L. kempii*, *L. olivacea* and *D. coriacea*.

Although there were four different groups with respect to δ^15^N values, there was broad interspecific overlap in trophic level. As a general rule of thumb, the discrimination factor from diet to consumer is ∼3–4‰, representing one trophic level (DeNiro & Epstein, 1981; Seminoff *et al.,* 2009, 2006). Taken together, our analyses of δ^13^C and δ^15^N values revealed that the trophic ecology of marine turtles is not as typological as has long been hypothesised based on life‐cycle patterns (Fig. 2) and trophic morphology (Fig. 3). While marine turtles exhibit some ecopartitioning of the marine realm, the patterns are far more complex owing to the substantial interspecific overlap of both their δ^13^C and δ^15^N values, and to tremendous intraspecific variation (Fig. 5). We now explore this intraspecific variation across the hierarchical levels described in Fig. 1.

### (2) Variation in trophic ecology among populations

In addition to the interspecific comparisons described above, our data set affords the most complete picture to date of intraspecific and inter‐population variation within each of several species (B in Fig. 1). However, it is possible that variation that might be ascribed to intraspecific variation in trophic ecology is really due to differences among basins in baseline isotope values (particularly ^15^N) which have been hypothesised to exist (McMahon, Hamady, & Thorrold, 2013; West *et al.,* 2009, but see Pethybridge *et al.,* 2018). Hence, we attempted to account for basin effects in several ways.

First, the nested ANOVAs that held tissue constant while testing for species differences within three ocean basins (Table S4b, d) revealed a basin effect on δ^13^C values, but not on δ^15^N values. Second, examination of the scatterplot of mean δ^13^C *versus* δ^15^N values (Fig. 5) reveals that values are not clustered by basin within species as would be expected if basin had an overriding effect. Rather it can be seen that high and low values within a species are often found within the same basin. Third, an additional ANOVA nesting basins within species showed no basin effect for δ^15^N values (Table S5d). Finally, we also attempted to adjust for inter‐basin differences in baseline levels of ^15^N using phytoplankton baseline δ^15^N values extracted from a recent study (Pethybridge *et al.,* 2018) (see Table S6, Fig. S3). This analysis did not materially alter the pattern shown in Fig. 5. Taken together, this lack of inter‐basin differences within these species indicates that the trophic ecology of a given species is not overly influenced by hypothesized differences among ocean basins in baseline δ^13^C and δ^15^N values (West *et al.,* 2009; McMahon *et al.,* 2013). A pattern of relatively similar ^15^N baseline levels across ocean basins, and ocean regions within basins has also been documented in a recent study of tuna species (Pethybridge *et al.,* 2018). Hence, we proceeded to evaluate intraspecific variation in isotope values as being truly reflective of species trophic ecology rather than being an artefact of basin effects.

The picture that emerges is that species exhibit tremendous intraspecific variation as evidenced by a broad range of δ^15^N values among populations for each species within each basin (Figs S2d, S3). Given that a discrimination factor of 3–4‰ is typically regarded as representing one trophic step (see Section I), it can be concluded that most species forage across two or more trophic levels. Of the species for which sufficient data exist (Table 2, Figs 5, S3), one species, *L. olivacea*, is likely to forage at only a single trophic level (Figs 5, S3). Two species, *C. mydas* and *E. imbricata*, are likely to forage at two trophic levels. SIAs have revealed cryptic diets in adult *C. mydas* with some populations being clearly omnivorous and others herbivorous, in contrast to the longstanding view that there is an obligate ontogenetic dietary shift from omnivory to herbivory resulting in adults being specialist herbivores (Hancock *et al.,* 2018; Hatase *et al.,* 2006, Figs 5, S3). Two species, *D. coriacea* and *C. caretta,* span more than two trophic levels between populations. Hence, δ^15^N values reveal not only more interspecific overlap than predicted from trophic morphology (see Section IV.1), but also considerable intraspecific variation in realised trophic levels not predicted by diet and trophic morphology.

Our conclusions regarding the causes of inter‐population differences are rather different from those drawn from single‐species case studies. Six studies have explored intraspecific differences in trophic ecology among geographically distinct populations of the same life stage. Four studies compared stable isotope ratios between conspecifics within the same life stage inhabiting different ocean basins: among *C. caretta* oceanic juveniles (Pajuelo *et al.,* 2010) and stranded juveniles, subadults, and adults (Tomaszewicz *et al.,* 2015); among *D. coriacea* oceanic adults (Wallace *et al.,* 2006); and among *E. imbricata* of unreported life stage (Moncada *et al.,* 1997). Two additional studies examined differences among populations of *C. caretta* and *C. mydas* within the same ocean basin (Vander Zanden *et al.,* 2013a *;* Cardona *et al.,* 2014).

Two of the between‐basin studies concluded that there is a basin effect whereas the other two did not. Pajuelo *et al*. (2010) and Wallace *et al*. (2006) found no differences in δ^13^C values in either *C. caretta* or *D. coriacea,* respectively, between two ocean basins. This similarity in carbon isotope ratio validates each species' inherent spatial foraging strategy, i.e. that they utilise the same macrohabitat in each basin. Both of these studies observed significantly enriched δ^15^N values in samples from the eastern Pacific. A difference in nitrogen isotope ratio typically indicates differences in trophic levels, but both studies present evidence that these observed differences might reflect differences in nitrogen‐cycling processes between the Atlantic and the eastern Pacific, rather than differences in trophic level.

By contrast, the study comparing different life stages *C. caretta* in the Atlantic and Pacific found higher δ^13^C values in the Atlantic, which was interpreted as reflecting differences in the spatial foraging strategy likely related to an ontogenetic switch (Tomaszewicz *et al.,* 2015). They found no difference in δ^15^N values between the two ocean basins. Examination of stable isotope patterns among different populations of *E. imbricata* in the western Pacific, southeastern Indian Ocean, and the Caribbean revealed higher δ^13^C values and lower δ^15^N values in the Caribbean population than in populations in the two other basins (Moncada *et al.,* 1997). The authors concluded that the δ^13^C values of the Caribbean population reflect a closer dependency on coral reefs, and the high δ^15^N values in the Pacific and Indian Ocean populations indicate a diet containing more non‐coral animal protein.

Two studies that have compared stable isotope composition between populations within the same ocean basin concluded that there are inter‐population differences within basins. The two studies examined the differences in isotopic niches among populations of *C. caretta* (Cardona *et al.,* 2014) and *C. mydas* (Vander Zanden *et al.,* 2013a) within the same ocean basin. In *C. caretta* in the Mediterranean, stable isotope composition represented a continuum that aligned with different foraging areas and their respective productivity levels. Oceanic currents and distance from the nesting beaches were hypothesized to be the drivers of the differences in foraging areas among populations (Cardona *et al.,* 2014). In *C. mydas* in the Caribbean, analyses revealed higher δ^15^N values in adult nesting females in Costa Rica compared to their foraging counterparts in Nicaragua, indicative of a potential omnivorous diet. Further investigations using amino acid‐compound specific isotope analysis (AA‐CSIA) revealed that the differences in stable isotope composition could be interpreted as a result of regional differences in primary production and differences in nutrient cycling, rather than evidence for an alternative foraging strategy between different populations (Vander Zanden *et al.,* 2013a).

The contrasting conclusions drawn from our meta‐analysis compared to these single‐species case studies concerning inter‐ or intra‐basin differences in baseline isotope values highlights the interpretive limitations inherent in two‐sample comparisons (Garland & Adolph, 1994). For example, a comparison of point samples from a single population in each basin could reflect basin differences (interpretations that have been made) but also differences in local conditions, which are not necessarily representative of the basin as a whole. While it is tempting to ascribe an observed difference to one possible cause over another in such comparisons, a two‐sample design does not permit such a distinction. For instance, several studies compared an eastern Pacific sample to an Atlantic sample. However, the eastern Pacific is substantially enriched in ^15^N compared to several other regions of the Pacific (Pethybridge *et al.,* 2018), and hence is not representative of the mean value in this basin. Because our meta‐analysis includes numerous observations from different regions in each of several basins, our analyses (Tables 2, 3, S4, S5, S6) yielded estimates of within‐basin variance that serve as a quantitative basis for between‐basin comparisons. In other words, we were able unambiguously to test the hypothesis that basins do not differ in their baselines, while accounting for any within‐basin variance that could obscure a true signal of variation in trophic ecology. Thus, our findings urge caution for future studies when interpreting heterogeneity in stable isotope values when the sampling design does not permit robust attribution among putative causes.

### (3) Variation in trophic ecology within populations

#### (a) *Variation in trophic ecology among life stages*

Complex life cycles are characterised by abrupt changes in trophic behaviour and habitat use, which result in shifts in trophic niche (Wilbur & Collins, 1973; Wilbur, 1980; Werner, 1988). This complexity is amplified in long‐lived organisms, which must balance an energetic trade‐off between maximising growth rates to minimise the time to maturity while minimising predation risk (Werner & Gilliam, 1984). This trade‐off often arises because different habitats vary in their productivity, which in turn influences local growth rates and time to maturity, but predation pressure is typically greatest in more productive habitats (Werner & Hall, 1988; Werner & Anholt, 1993). Body size throughout ontogeny plays a major role in resolving this trade‐off, because of its large influence on an organism's energetic requirements and ability to exploit resources, but also its susceptibility to natural enemies (Werner & Gilliam, 1984; Werner & Hall, 1988; Werner & Anholt, 1993). It can also be a factor in reducing resource competition between life stages (Wilbur, 1980; Werner & Gilliam, 1984).

Not surprisingly, the complex life cycles and longevity of marine turtles produce complicated patterns of habitat use and trophic ecology across ontogeny (C.1 in Fig. 1). This complexity arises due to both a progression of sizes (hatchlings grow more than two orders of magnitude before attaining maturity), and age‐associated differences in form, function, and ecology (different life stages use different macrohabitats; Fig. 2).

There is a persistent knowledge gap concerning the habitat use and specific spatial trophic ecology of early life‐history stages in turtles because they are hard to observe directly. Before the use of SIA, knowledge of ontogenetic patterns in the trophic ecology of early life stages was derived from studies using methods such as mark–recapture and gut‐content analysis (Bjorndal, 1997). Such studies provided the initial evidence for an ontogenetic shift from oceanic to neritic habitat, thus establishing the existence of the Type 2 life cycle (Fig. 2). Yet it has remained difficult to pinpoint the size class or age at which the transition from oceanic to neritic feeding habitats occurs. Additionally, in the case of *C. mydas* the gut‐content approach revealed a shift in diet from carnivory to herbivory, across a certain body‐size threshold (Bjorndal & Bolten, 1988; Bjorndal, 1997; Bolten, 2003). As an example, Bjorndal & Bolten (1988) detected a shift from oceanic to neritic habitats at 20–25 cm curved carapace length (CCL) in *C. mydas* in the northwestern Atlantic using repeated measurements of individuals and morphometric analysis. These findings of ontogenetic niche shifts raise two specific questions about the trophic ecology of different life stages. The first concerns the timing (and size) of the predicted transition from oceanic to coastal areas. The second concerns the composition of diet (possibly cryptic) and trophic level of individuals at a given life stage.

Stable isotopes have provided a powerful tool to address these questions about habitat use and diet composition of marine turtle early life stages. By analysing stable isotopes in inert tissues (i.e. bone or scute layers), resampling individuals multiple times, or combining SIA with skeletochronology, SIA permits assessment of an individual's foraging history over multiple years or even its entire life. To date, 46 studies have investigated ontogenetic differences in habitat and diet in *C. caretta* (*N* = 16), *C. mydas* (*N* = 29), *D. coriacea* (*N* = 1), *E. imbricata* (*N* = 2), *L. kempii* (*N* = 1), and *L. olivacea* (*N* = 2) (Table S3).

Several stable isotope studies have addressed the question concerning the timing of ontogenetic shifts from oceanic to neritic habitat. One approach has been to examine stable isotope values in different bone growth layers and translate this into an estimation of size classes in *C. caretta* (Snover *et al.,* 2010) and *C. mydas* (Howell *et al.,* 2016; Velez‐Rubio *et al.,* 2016). Yearly somatic growth is recorded in annual marks in humeri cross sections, and a transition from narrow growth marks to wider growth marks indicates a sharp increase in growth rates and a potential shift from oceanic to neritic habitats. SIA of the different layers corroborates a habitat shift congruent with pelagic *versus* benthic feeding, and the number of annual growth marks reveals the age at which the habitat shift occurred.

A second approach has been to attempt to transform size classes into age estimates. In north‐eastern Atlantic *C. caretta,* two studies estimated the shift to occur with a straight carapace length (SCL) of about 54–55 cm, at ∼12 years of age (Avens *et al.,* 2013; Ramirez *et al.,* 2015). In Atlantic *C. mydas*, Reich, Bjorndal, & Bolten (2007) estimated the transition from oceanic to neritic habitats to occur at 3–5 years, at ∼25–35 cm SCL. To date, no stable isotope studies have examined habitat shift in *L. kempii*, the most endangered marine turtle species.

SIAs have corroborated the initial findings of an ontogenetic habitat shift and have provided age estimates. However, SIA has also revealed greater complexity than was previously appreciated. One new insight is that the behavioural flexibility required to shift habitats appears to be confined to immature stages. Once maturity is attained, adults seemingly have a diminished capacity to switch foraging habitat preferences (oceanic *versus* neritic), even if they are using a habitat that is sub‐optimal in resource abundance (Cardona *et al.,* 2017). A second insight gained from recent SIA is that there is inter‐individual variation among juveniles within a single population in the timing and rapidity of such transitions (Ramirez *et al.,* 2015). Some juveniles shift quickly and discreetly (within a year), while shifts in others are more protracted (up to 5 years) and happen in increments. Further, in some populations juveniles also display a recurrent seasonal (winter *versus* summer) shift between neritic and oceanic foraging habitats (McClellan *et al.,* 2010). Further, some individuals never shift and remain in the oceanic habitat (Cardona *et al.,* 2017). Finally, several studies have demonstrated between‐population variation in use of oceanic and neritic foraging grounds in both *C. caretta* (Casale *et al.,* 2008; McClellan *et al.,* 2010) and *C. mydas* (Hatase *et al.,* 2006; Araujo Morais *et al.,* 2014).

There are multiple reasons why marine turtles do not remain in one developmental habitat until they reach maturity. Complex life‐cycle theory postulates that different habitats utilised for foraging by different life stages play an important role in growth and maturation and might obviate intraspecific competition between life stages (Wilbur & Collins, 1973; Werner, 1988). In marine turtles, the open ocean provides protection from predators and thermal refuges for small size classes associated with floating *Sargassum* (Witherington, Hirama & Hardy, 2012). Also, predator densities are lower in the open ocean (Carr, 1987; Bolten, 2003). However, there will be a trade‐off with slower growth rates in oceanic habitat because productivity is lower than in coastal areas. Once a size refuge from predation is attained, juveniles can exploit the more productive coastal foraging areas, which accelerates growth (Bolten, 2003).

The second question that SIA has illuminated regards ontogenetic shifts in diets, such as trophic level and dietary composition (e.g. herbivory *versus* carnivory), which are unrelated to changes in spatial habitat use (shift from oceanic pelagic prey to neritic benthic prey). Four studies have investigated this question in *C. caretta* in Atlantic and Indian Ocean populations (Wallace *et al.,* 2009; McClellan *et al.,* 2010; Thomson *et al.,* 2012; Hall *et al.,* 2015); 19 studies in Atlantic *C. mydas* (Burgett *et al.,* 2018; Cardona *et al.,* 2009b; Di Beneditto, Siciliano & Monteiro, 2017; Gillis *et al.,* 2018; Gonzales Carman *et al.,* 2014; Hancock *et al.,* 2018; Howell *et al.,* 2016; Velez‐Rubio *et al.,* 2016; Williams *et al.,* 2014), Pacific *C. mydas* (Arthur, 2008; Barceló, 2018; Lemons *et al.,* 2011; Prior, Booth & Limpus, 2015; Rodríguez‐Barón, 2010; Sampson *et al.,* 2017; Santos‐Baca, 2008; Shimada *et al.,* 2014), and *C. mydas* in the Indian Ocean (Burkholder *et al.,* 2011) and in the Mediterranean (Cardona *et al.,* 2010); one study in Atlantic *D. coriacea* (Wallace *et al.,* 2014), one study in *E. imbricata* (Ferreira *et al.,* 2018), and one study in *L. olivacea* (Peavey *et al.,* 2017).

No differences in diet and trophic level among life stages have been detected in stable isotope ratios of *C. caretta* despite several attempts to find them (Wallace *et al.,* 2009; McClellan *et al.,* 2010; Thomson *et al.,* 2012; Hall *et al.,* 2015). The only differences were those congruent with the already mentioned spatial habitat shift and a resulting increase in trophic level with body size when shifting from pelagic to benthic prey. The overall pattern indicated by SIA of *C. caretta* is that observed changes in stable isotope compositions are based solely on the transition of juveniles between macrohabitats rather than on a change in trophic level (McClellan *et al.,* 2010; Hall *et al.,* 2015).

By contrast, in *D. coriacea* an increase in δ^15^N values with increasing body size was detected among life stages (Wallace *et al.,* 2014) and in *E. imbricata* data showed that immature life stages occupy a significantly smaller isotopic niche than adults, but no difference in trophic level was found (Ferreira *et al.,* 2018). Several SIA studies of *C. mydas* from multiple ocean basins indicate a general pattern of ontogenetic dietary shift to a lower trophic level with increasing body size: from carnivory or omnivory to herbivory with increasing body size. For example, Velez‐Rubio *et al*. (2016) documented a relationship between diet (a shift between omnivory and herbivory) and body size (gelatinous macrozooplankton in turtles <45 cm CCL, and predominantly herbivory in individuals >45 cm). Another study found a similar pattern, but at a larger body size (CCL >59 cm) (Cardona, Aguilar & Pazos, 2009a). These findings corroborate earlier work based on gut‐content analyses indicating a transition from omnivory in early life stages to strict herbivory in adults (Bjorndal, 1997). However, a study in an eastern Pacific population did not find differences in diet between adults and immature stages suggesting a lack of ontogenetic dietary shift and that adults remained omnivores (Lemons *et al.,* 2011). Studies in the western Pacific and eastern Atlantic found a similar pattern (Shimada *et al.,* 2014; Hancock *et al.,* 2018). Further, one study detected an asynchronous shift between diet and which dietary components constitute the main nutritional source (protein *versus* plant matter) (Cardona *et al.,* 2010). A high‐protein diet derived from carnivory fuels the growth of early life stages, thus minimising time to maturity and attainment of a size refuge from predation (Werner & Gilliam, 1984; Werner, 1988; Werner & Hall, 1988; Werner & Anholt, 1993). Additionally, several studies found regional differences (Prior, Booth, & Limpus, 2015; Gillis *et al.,* 2018) and inter‐individual differences in diet within the same life stage (Barceló, 2018; Burgett *et al.,* 2018).

#### (b) *Variation in trophic ecology between sexes*

The next hierarchical level at which a species may mitigate intraspecific competition is between sexes of adult individuals (C.2 in Fig. 1). This question has barely been investigated in marine turtles, which reflects a persistent knowledge gap concerning male biology and ecology because of a research bias towards studying nesting females. Only seven studies to date have investigated intersexual differences in trophic ecology: *C. caretta* in the Atlantic (Pajuelo *et al.,* 2016, 2012), *D. coriacea* in the Atlantic (Dodge, Logan & Lutcavage, 2011; Wallace *et al.,* 2014), *C. mydas* in the Atlantic and in the Pacific (Vander Zanden *et al.,* 2013a *;* Prior *et al.,* 2015), and *L. olivacea* in the Pacific (Peavey *et al.,* 2017).

Only one study detected significant intersexual differences in stable isotope composition, particularly in δ^13^C, in *D. coriacea* (Dodge, Logan, & Lutcavage, 2011). These data suggest differences in spatial foraging patterns, which could be the result of divergent migratory cycles between male and female *D. coriacea* that reside for different time intervals in northern foraging areas (James, Eckert, & Myers, 2005a; James, Myers & Ottensmeyer, 2005b), with males spending annually extended periods in tropical, coastal areas adjacent to nesting beaches, and females foraging for 2–3 years in northern oceanic habitat. The study found elevated female δ^13^C and δ^15^N values compared to males, which suggests that females forage closer to the coast or at lower latitudes than males (Kelly, 2000; Rubenstein & Hobson, 2004). However, the reverse pattern should be expected for male and female stable isotope ratios according to their divergent migratory cycles. An alternative explanation could be that the energetic demands of nesting (migration, egg production, starvation during nesting season) and the resulting nutritional stress cause elevated δ^13^C and δ^15^N values in females (Hobson, Alisauskas & Clark, 1993).

By contrast, all other studies comparing male and female trophic ecology did not detect any isotopic differences in either spatial foraging patterns or trophic level. The most plausible explanations for this is that first, both sexes of marine turtles exhibit natal philopatry and it is likely that they will also share the same developmental habitats and later on foraging habitat. Further, they exhibit very little sexual size dimorphism (Figgener, Bernardo & Plotkin, 2018) compared to other turtle species (Abouheif & Fairbairn, 1997; Agha *et al.,* 2018; Berry & Shine, 1980; Bonnet *et al*., 2010; Ceballos *et al.,* 2013; Gosnell, Rivera & Blob, 2009; Halámková, Schulte & Langen, 2013), or species where strong size dimorphism aligns with divergence in trophic morphology and ecology (e.g. lizards and bird‐eating hawks (Schoener, 1967, 1984). Lastly, the energy expenditures between the two sexes are similar and would not suggest a difference in diet or trophic level. Female marine turtles bear the energetic expenditure of egg production, however in most species (excepting *Lepidochelys* spp.) females counterbalance these expenditures by skipping nesting seasons to forage for extended periods (Limpus, 1993; Miller, 1997; Plotkin, 2003; James, Myers, & Ottensmeyer, 2005b), whereas males migrate to breeding sites adjacent to nesting beaches annually (Limpus, 1993; James *et al.,* 2005a; Hays *et al.,* 2010).

While most of the available data indicate no differences in male–female trophic ecology, this conclusion should be viewed as tentative, given the dearth of data and that differences in migratory timing between sexes (males spending time annually in coastal, neritic areas), in combination with the protracted integration times of stable isotopes into tissues, could result in differences in at least δ^13^C values. Future studies of multiple populations of multiple species should attempt to integrate male–female comparisons.

### (4) Variation in trophic ecology among adults within a population and its effect on individual fitness

The last hierarchical level at which intraspecific competition might be ameliorated is among individuals irrespective of ontogenetic stage and sex (D in Fig. 1). This level of variation is surprisingly understudied, although it is an emerging theme in recent literature (Bolnick *et al.,* 2003; Araujo, Bolnick, & Layman, 2011; Violle *et al.,* 2012). Interestingly, this question has been studied extensively in adult marine turtles using SIA: 41 studies have investigated variation in trophic ecology among individuals within populations for six species and two ocean basins (Table S3). The two main patterns emerging from these SIA studies are first that most populations comprise two or three subgroups that exhibit consistent associations with geographically distinct foraging areas and second, that populations exhibit high inter‐individual variation in trophic ecology.

The first pattern typically involves a spatial subdivision of adults foraging in either highly productive or low‐productivity habitats. This dichotomy often aligns either with neritic *versus* oceanic foraging areas (Eder *et al.,* 2012; Hatase, Omuta & Tsukamoto, 2010; Hatase, Omuta & Tsukamoto, 2013; Hawkes *et al.,* 2006; Lopez‐Castro *et al.,* 2013; Robinson *et al.,* 2016; Watanabe *et al.,* 2011) or high‐ *versus* low‐latitude foraging areas (Ceriani *et al.,* 2012). Interestingly, *L. olivacea*, which is known for its long‐distance, nomadic migrations (Plotkin, 2010), does not show a dichotomy between individuals feeding in high‐ or low‐productivity habitats (Dawson, 2017; Peavey *et al.,* 2017; Petitet & Bugoni, 2017). The presence of divergent spatial foraging strategies within populations and the exact patterns vary among the species and populations examined. But divergent spatial foraging strategies have been recorded in populations of four out of six studied species (*C. caretta*, *C. mydas*, *E. imbricata*, and *D. coriacea*).

Although a dichotomous foraging strategy within a population is common in some species of marine turtles, its underlying mechanisms are barely studied. One attempt to determine whether foraging dichotomies have a genetic basis concluded that they are the result of phenotypic plasticity cued by early growth rates (Hatase, Omuta, & Tsukamoto, 2010; Watanabe *et al.,* 2011). An understanding of the causes of divergent foraging strategies warrants further examination.

An observation made by several studies is that individuals foraging in more productive areas (e.g. neritic) tend to have a larger body size compared to those foraging in less‐productive areas (Hatase *et al.,* 2002; Eder *et al.,* 2012; Lontoh, 2014; Vander Zanden *et al.,* 2014a *;* Patel *et al.,* 2015). Additionally, in northeastern Atlantic *C. caretta*, data indicate that head size in adults is related to preferred foraging areas and not to trophic level and is only to a small degree explained by variation in body size (Price *et al.,* 2017).

There have been several attempts to evaluate the implications of foraging dichotomies on fitness. The most comprehensive effort to date examined the foraging dichotomy (neritic *versus* oceanic) among sympatrically nesting *C. caretta* in the Western Pacific detected using SIA (Hatase, Omuta, & Tsukamoto, 2013). Using a remarkable long‐term data set of 26 years, the authors analysed variation in different life‐history traits and found significant differences between the two foraging groups in body size, clutch size, clutch frequency, breeding frequency, and remigration intervals. Using this information they computed cumulative reproductive output (total number of emerged hatchlings produced per female) of the two foraging groups and found a significant difference between foraging groups, with neritic feeders having a 2.4‐fold larger reproductive output. Several other studies of *C. caretta* (Hatase *et al.,* 2002; Eder *et al.,* 2012; Cardona *et al.,* 2014; Vander Zanden *et al.,* 2014a *;* Patel *et al.,* 2015 *;* Ceriani *et al.,* 2017) and *D. coriacea* (Lontoh, 2014) also investigated this question, but with short‐term data (usually a single nesting season) and only for a few traits (usually body size and clutch size). All of these studies detected similar life‐history differences between individuals using high‐ *versus* low‐productivity foraging areas. Although these findings are congruent with Hatase *et al*.'s (2013) findings that individuals using high‐productivity foraging areas have higher fitness than individuals feeding in low‐productivity areas, these other studies should be viewed as preliminary because they only represent snapshots of fitness components. Robust conclusions that the dichotomous foraging strategies that have been repeatedly identified using SIA translate into fitness consequences can only be drawn with long‐term data.

The compelling finding of apparent fitness differences between foraging groups (Hatase *et al.,* 2013) raises the further question of whether a trade‐off exists that balances fitness between the two strategies, therefore maintaining both within a single population. To address this question numerous traits including age (Hatase *et al.,* 2010), egg size & components (Hatase, Omuta & Komatsu, 2014), hatchling size (Hatase, Omuta & Komatsu, 2015) and various traits presumed to be indicative of offspring quality (Hatase *et al.,* 2018) have been investigated that might contribute to such a trade‐off. None of these studies revealed a fitness trade‐off. A robust way to evaluate the fitness effects of divergent life‐history strategies is a life‐table approach, which can mathematically determine whether alternative strategies produce equivalent fitness (Tilley, 1980). This approach requires age‐specific data on onset of reproduction, fecundity, survivorship, and the duration of the reproductive lifespan. Such analyses are not currently feasible for marine turtles because of a lack of suitable data for a single species, let alone for the divergent population‐level foraging subgroups identified by SIA.

The second emerging pattern is that many populations show high inter‐individual variability that does not align with geographically distinct foraging areas. Although 61% of the available studies concern *C. caretta*, this pattern has been identified in four out of six species studied (*C. caretta*, *C. mydas*, *L. kempii*, *L. olivacea*) and aligns with the findings of our meta‐analysis (Section IV.1). Often individuals within a population are more specialised, that is, individuals have a narrower isotopic niche width than the average isotopic niche width of the population or species would suggest (Pajuelo *et al.,* 2016; Peavey *et al.,* 2017; Petitet & Bugoni, 2017; Reich *et al.,* 2017; Vander Zanden, Bjorndal & Bolten, 2013b; Vander Zanden *et al.,* 2010).

Further, where studied, this among‐individual sub‐specialisation in adults is persistent through time (Pajuelo *et al.,* 2016; Vander Zanden, Bjorndal, & Bolten, 2013b, 2010). These chronological records have been obtained by either looking at annual growth layers in bone or scute tissue, or by resampling of recaptured individuals over time. For instance, Vander Zanden *et al*. (2010) detected long‐term specialisation in resource use of individual *C. caretta* by examining stable isotope composition across numerous scute layers reflecting up to 12 years of foraging history. Thus marine turtles add to the growing literature that demonstrates that generalist animal species are often composed of ecologically heterogeneous individuals that repeatedly differ in foraging behaviour and use different subsets of the available resources (Bell, Hankison & Laskowski, 2009; Bolnick, Svanback & Araujo, 2007a; Bolnick *et al.,* 2003).

These studies provide evidence for higher intraspecific variation in the exploitation of the trophic axis than previously recognised, thus indicating that individualism is an important component of marine turtle trophic ecology.

## V. DISCUSSION

This systematic review was motivated by the lack of synthesis of marine turtle stable isotope data to achieve a more in‐depth view of their ecology and evolution. This exercise revealed far greater complexity in trophic ecology within and among species than previously hypothesised. These findings inform marine turtle ecology, conservation and management, elucidate the ecological role of marine turtles in the marine realm, and have much broader implications for the study of ecological radiations.

### (1) Novel insights about marine turtle trophic ecology from stable isotope analysis

Marine turtles are widely distributed throughout all ocean basins and inhabit diverse ecosystems, and it has long been appreciated that they show a clear interspecific signature of ecopartitioning of the marine realm and are highly diversified in life‐history traits and ecology (Hendrickson, 1980; Van Buskirk & Crowder, 1994; Bjorndal, 1997; Bjorndal & Jackson, 2002; Bolten, 2003). They show particularly striking variation in trophic morphology, which is evident among both extant species (Fig. 3) and throughout the rich fossil record spanning more than 120 million years (Kear & Lee, 2006; Parham & Pyenson, 2010; Cadena & Parham, 2015; Gentry, 2017).

Despite these long‐standing qualitative characterisations of variation in marine turtle trophic ecology, our meta‐analysis of interspecific variation in isotopic composition is the first quantitative assessment of the hypothesis that they do partition marine resources, and the extent to which species differ (Figs. 4, 5, S2). Our quantitative analysis corroborates previous but incomplete qualitative evidence from variation in trophic morphology, microhabitat use and gut‐content analyses that marine turtles exhibit ecopartitioning of resources. No prior study has performed any quantitative statistical assessment of this hypothesis, in part because neither quantitative characterisation of trophic morphology, nor of habit use, nor of gut contents across all species has ever been published.

Additionally, our review revealed a continuum of trophic sub‐specialisation in most species, which extends beyond interspecific differences and ranges from variation in trophic niches between populations of the same species in different ocean basins and geographic regions, to variation of trophic niches among life stages and individuals within populations (Fig. 1). This ubiquity of trophic sub‐specialisation at many levels exposes a far more complex view of marine turtle ecology and resource‐axis exploitation than is suggested by species diversity alone.

While our review has demonstrated the power of SIA to elucidate many aspects of trophic ecology of marine turtles, it has also revealed substantial research gaps. These gaps probably exist because most studies were typically addressing narrower questions concerned with conservation, usually focusing on a single species. In particular, we note three major issues. First, while most species occupy multiple ocean basins (Table S1), there is uneven sampling across ocean basins (Table S2) and regions within basins (Figgener *et al.,* 2019). For instance, only five studies have been conducted in the Indian Ocean (Table S2) (Moncada *et al.,* 1997; Burkholder *et al.,* 2011; Thomson *et al.,* 2012, 2018; Robinson *et al.,* 2016) and none in the Red Sea. This is relevant because heterogeneity of biogeochemical processes on both the basin and regional scales (Wallace *et al.,* 2006; Pethybridge *et al.,* 2018) affects baseline values of δ^13^C and δ^15^N, and populations are different in size and face different intensities of threats.

The second issue is that sampling effort for each species is uneven. For instance, there is a paucity of studies of *L. kempii*, *E. imbricata*, and *L. olivacea* compared to *C. caretta*, *C. mydas*, and *D. coriacea* and no studies for *N. depressus* (Table S2).

The third issue is that there is no common currency or standardisation for sampled tissues across stable isotope studies, hindering comparative analyses. Nearly a dozen different tissues have been used in SIA of marine turtles (Table 2 in Figgener *et al.,* 2019), but tissues differ in discrimination factors and turnover times (Reich *et al.,* 2008; Seminoff *et al.,* 2009, 2006; Vander Zanden *et al.,* 2012, 2014b), which both influence stable isotope estimates. Moreover, in most cases, there is no way to convert stable isotope values of one tissue accurately into the values of another (Ceriani *et al.,* 2014; Vander Zanden *et al.,* 2014b). Hence, we suggest that future studies always include stable isotope estimates from skin, a tissue easily sampled and stored, which will facilitate future comparative analyses.

In conclusion, our comparative analysis indicates that the longstanding idea that trophic morphology provides robust insights into interspecific variation in foraging ecology is incomplete, both with respect to marine turtles and possibly in other vertebrate radiations as well. In other words, SIA is a powerful tool to detect cryptic variation in trophic ecology beyond trophic morphology, permitting a more comprehensive understanding of ecological radiations and food‐web structure.

### (2) Implications for marine turtle conservation and management

The continuum of trophic specialisation both among and within species of marine turtles revealed by our review has several implications for conservation and management. First, the ubiquity of this pattern adds another underappreciated dimension to marine turtle conservation and management beyond that informed by traditional genetically defined management units. In particular, it is now clear that even within management units marine turtle populations are comprised of individuals using ecologically distinct strategies and therefore are not ecologically exchangeable. Ecological exchangeability refers to the idea that individuals can be moved between populations and can occupy the same ecological niche or selective regime (Crandall *et al.,* 2000). Under this idea, the null hypothesis is that two or more populations of a species are ecologically equivalent, even if they are genetically distinct.

Conversely, two or more populations that are ecologically distinct are not ecologically exchangeable, even if they are part of the same genetically defined management unit. Numerous studies reviewed here show that the latter is typically the case for marine turtles. This ecological diversity within stocks necessitates a more comprehensive management approach beyond the genetic stock concept, which drives current understanding of management units. Related to this issue is the fact that research effort across genetically defined management units is uneven, and many stocks are completely unstudied (Pearson *et al.,* 2017). Hence, there could be as‐yet‐unrecognised cryptic variation in trophic ecology within these units.

Another consideration is that the two most geographically restricted species are essentially unstudied with respect to stable isotopes. *Natator depressus*, which is listed as data deficient by the IUCN (Red List Standards & Petitions Subcommittee, 1996), has yet to be studied, and there are only two studies of *L. kempii*, one of the two most endangered marine turtle species (Marine Turtle Specialist Group, 1996; Plotkin, 2016). The insights concerning trophic ecology (habitat use, trophic level) that would emerge from SIA of these species would be an invaluable tool for their conservation and management.

Another insight from our review is that SIA has revealed previously unknown patterns of habitat use of early life stages of marine turtles, which are poorly studied because direct observations of foraging areas in the marine realm are logistically challenging. For example, time series sampling of humeri of stranded turtles (Tomaszewicz *et al.,* 2016) has yielded insights into ontogenetic patterns in trophic ecology (Tomaszewicz *et al.,* 2018, 2017b), yet few studies to date have exploited this opportunity. Such insights would facilitate the location of critical habitats for growth and development of juveniles and subadults, potentially resulting in more effective protective measures for these life stages Additionally, locating hotspots of immature life stages would likely increase our ability to study them directly using mark–recapture and tracking studies to close longstanding gaps in our understanding of marine turtle demography.

### (3) Ecological roles of marine turtles in the marine realm

The ubiquitous signal of ecological versatility among and within marine turtle species revealed by our synthesis paints a more complex picture of their ecological roles in the marine realm than has previously been appreciated and which is distinct from those of other large, marine predatory vertebrates. It is now widely documented that losses of apex predators, including in marine systems, cause a wide variety of down‐web effects including trophic cascades and general trophic downgrading of marine ecosystems (Pace *et al.,* 1999; Heithaus *et al.,* 2008; O'Gorman & Emmerson, 2009; Estes *et al.,* 2011), secondary extinctions (Borrvall & Ebenman, 2006), altered biogeochemical cycles (Estes *et al.,* 2011), and regime shifts (Scheffer *et al.,* 2001; Barnosky *et al.,* 2012). Like other large marine predatory vertebrates, all species of marine turtles are of conservation concern, but the justification for their conservation is largely driven by their charismatic appeal rather than because of their ecological role in marine ecosystems (but see Bjorndal & Jackson, 2002).

Perhaps the most remarkable finding to emerge from our meta‐analysis is that adults of four species of marine turtles exhibit broad intraspecific trophic niches, foraging across 2–4 trophic levels among and within populations [*C. caretta*, *D. coriacea*, *E. imbricata*, *L. olivacea* (Table 2, δ^15^N axis on Fig. 5; Figgener *et al.,* 2019)]. This pattern is also evident within a single study of a fifth species, *L. kempii* (Reich *et al.,* 2017). These broad trophic niches of marine turtle are unlike those found in other marine predators. SIA of marine predators as varied as squid (Navarro *et al.,* 2013), bony fishes (Torres‐Rojas *et al.,* 2014; Pethybridge *et al.,* 2018), sharks (Estrada *et al.,* 2003; Hernandez‐Aguilar *et al.,* 2016), and cetaceans (Abend & Smith, 1997; Hooker *et al.,* 2001; Herman *et al.,* 2005) consistently exhibit a narrow isotopic niche indicating feeding at a single, usually high, trophic level. What is even more remarkable is that this pattern of feeding across multiple trophic levels occurs in three species that otherwise exhibit trophic specialisations for certain types of prey –*D. coricaea* (gelativory), *C. caretta* (durophagy) and *E. imbricata* (spongivory). Thus, marine turtle species span a broader ecological continuum in the oceans far beyond that suggested by their well‐established trophic ecomorphology, showing a ubiquitous ecological versatility.

This ecological versatility is also evident intraspecifically, both across ontogeny and among individuals (Fig. 1). Although these questions have only been addressed in two species (*C. caretta* and *C. mydas*), several insights have emerged. Where studied, juveniles appear to be more flexible in their dietary choices and foraging habitat use, whereas adults are typically consistent in both respects through time (Section IV.3*a* and IV.4). A second insight is that oceanic and neritic juveniles exhibit less individual specialisation in trophic ecology than adults. Additionally, in some species, adults exhibit different but individually consistent foraging strategies, thus resulting in a generalist population with individual specialists. Thus, both ontogenetic variation and individuality in foraging strategies expand the trophic footprint of marine turtles. Analysis of ontogenetic and inter‐individual variation in trophic ecology of other species is likely to be a fruitful area for future research.

Taken together, the inter‐ and intraspecific signal of marine turtle feeding across numerous trophic levels indicates a complex interconnectedness with an influence upon marine food webs. Theoretical and empirical studies of food‐web connectedness generally indicate that such multilevel trophic interactions act to stabilise food webs (Dunne, Williams & Martinez, 2002; O'Gorman & Emmerson, 2009; Thébault & Fontaine, 2010), buffering their dynamics against species gains and losses. By contrast, food webs tend to become destabilised when species that feed on a single trophic level are gained or lost. For example, losses of apex predators have been shown to produce trophic cascades across both aquatic and terrestrial ecosystems (Pace *et al.,* 1999; Heithaus *et al.,* 2008; O'Gorman & Emmerson, 2009; Estes *et al.,* 2011; Ripple *et al.,* 2014) and in the extreme, may result in down‐web extinctions (extinctions at lower trophic levels) (Borrvall & Ebenman, 2006; Sanders *et al.,* 2018; Säterberg, Sellman & Ebenman, 2013). Hence, that marine turtles feed across multiple trophic levels, both inter‐ and intraspecifically, indicates that they likely have a stabilising effect on food webs buffering trophic cascades that are elicited by the removal of apex predators such as sharks (Heithaus *et al.,* 2008). This broader view of the ecological role of marine turtles in the marine realm also provides a material argument for their conservation beyond their charismatic appeal. Additionally, this broad ecological role of marine turtles indicates that they may be among the best sentinels of ocean health, reflecting changes in baseline primary productivity and nitrogen‐cycling processes transferred through several trophic levels (Wallace *et al.,* 2006).

### (4) Implications for future research on ecological radiations

Although marine turtles are well‐known subjects of conservation efforts, their value as a model system for understanding broader ecological and evolutionary questions is underappreciated. In particular, the trophic complexity within and among species revealed by our analyses suggests that novel insights concerning resource partitioning in other ecological radiations might arise from SIAs across the hierarchical levels described in Fig. 1.

Since Darwin first remarked upon the striking variation in trophic morphology among *Geospiza* finches (Darwin, 1839), analyses of easily recognisable interspecific differences in body size and sizes and shapes of trophic structures have been the dominant theme in studies of ecological radiations (Schluter, 2000; Streelman & Danley, 2003). This research tradition has demonstrated that trophic morphology can reliably predict some degree of interspecific ecopartitioning, but it also has shown that ecopartitioning is often imperfect.

Because SIA is a measure of realised trophic ecology, it gives a different and more comprehensive perspective on the degree to which closely related species partition *versus* overlap in resource use than trophic morphology alone. Some insights that have emerged from SIA are broader trophic niches in the case of aquatic insect ecomorphs (see Section I), and both cryptic habitat use and diet breadth as we have shown here for marine turtles. Thus, because the degree of overlap predicted by trophic morphology underestimates the true breadth of realised trophic niche, future studies of ecological radiations would likely benefit from incorporation of SIA, advancing beyond the singular consideration of trophic morphology.

## VI. CONCLUSIONS

(1) Our contribution aimed to provide a quantitative analysis of interspecific variation and a comprehensive review of intraspecific variation in trophic ecology of marine turtles across different hierarchical levels, marshalling insights about realised trophic ecology derived from stable isotopes.

(2) Our study reveals a more intricate hierarchy of ecopartitioning by marine turtles than previously recognised based on trophic morphology and dietary analyses. We found strong statistical support for interspecific partitioning, as well as a continuum of intraspecific trophic sub‐specialisation in most species across several hierarchical levels beyond interspecific differences. This ubiquity of trophic sub‐specialisation at many levels exposes a far more complex view of marine turtle ecology and resource‐axis exploitation than is suggested by species diversity alone.

(3) Our findings are highly relevant to conservation management because they imply ecological non‐exchangeability, which introduces a new dimension beyond that of genetic stocks which drives current conservation planning.

(4) The insight that marine turtles are robust sentinels of ocean health and likely stabilise marine food webs has broader significance for studies of marine food webs and trophic ecology of large marine predators.

(5) The value of marine turtles as a model system for understanding broader ecological and evolutionary questions is underappreciated and our findings have broader implications for the study of ecological radiations. Particularly, the unrecognised complexity of ecopartitioning beyond that predicted by trophic morphology suggests that this dominant approach in adaptive radiation research likely underestimates the degree of resource overlap and that interspecific disparities in trophic morphology may often over‐predict the degree of realised ecopartitioning. Hence, our findings suggest that stable isotopes can profitably be applied to study other ecological radiations and may reveal trophic variation beyond that reflected by trophic morphology.

## Supporting information


**Table S1.** A survey of studies documenting regional spatial overlap of marine turtle species.
**Table S2.** Summary table showing the number of studies using stable isotope analysis (SIA) of δ^13^C and δ^15^N to investigate the trophic ecology of marine turtles, organised by species and ocean basin.
**Table S3.** Summary table showing the number of studies using stable isotope analysis (SIA) of δ^13^C and δ^15^N to investigate the trophic ecology of marine turtles, organised by species and broader study topic introduced in the conceptual model shown in Fig. 1.
**Table S4.** Nested analyses of variance (ANOVAs) modelling interspecific differences in stable isotope values taking into account variation among sampled tissues and ocean basins.
**Table S5.** Nested analyses of variance (ANOVAs) modelling difference in stable isotope values among basins and tissues taking into account variation among species.
**Table S6.** Summary statistics of unadjusted δ^15^N and adjusted δ^15^N values from 91 data points of adult marine turtles used in our meta‐analysis.
**Figure S1.** High‐resolution version of the images shown in Fig. 3.
**Figure S2.** Exploratory data analyses comparing values of δ^13^C and δ^15^N among species within tissues within one ocean basin (Atlantic, the basin with most estimates) (A, C) and among species within ocean basins within one tissue (skin, the tissue with most estimates) (B, D).
**Figure S3.** Scatterplot of 91 means from values of δ^13^C and adjusted values of δ^15^N [adjusted using baseline phytoplankton data extracted from Pethybridge *et al.,* 2018, see Table S6] in adults of six marine turtle species.
