# Supplementary material for: Beyond trophic morphology: stable isotopes reveal ubiquitous versatility in marine turtle trophic ecology
Source: Biol Rev Camb Philos Soc. 2019 Jul 24;94(6):1947–73. doi: 10.1111/brv.12543 (PMC6899600; doi:10.1111/brv.12543)
Supplement: Supplementary file 1 — Table S1. A survey of studies documenting regional spatial overlap of marine turtle species. Table S2. Summary table showing the number of studies using stable isotope analysis (SIA) of δ13C and δ15N to investigate the trophic ecology of marine turtles, organised by species and ocean basin. Table S3. Summary table showing the number of studies using stable isotope analysis (SIA) of δ13C and δ15N to investigate the trophic ecology of marine turtles, organised by species and broader study topic introduced in the conceptual model shown in Fig. 1. Table S4. Nested analyses of variance (ANOVAs) modelling interspecific differences in stable isotope values taking into account variation among sampled tissues and ocean basins. Table S5. Nested analyses of variance (ANOVAs) modelling difference in stable isotope values among basins and tissues taking into account variation among species. Table S6. Summary statistics of unadjusted δ15N and adjusted δ15N values from 91 data points of adult marine turtles used in our meta‐analysis. Figure S1. High‐resolution version of the images shown in Fig. 3. Figure S2. Exploratory data analyses comparing values of δ13C and δ15N among species within tissues within one ocean basin (Atlantic, the basin with most estimates) (A, C) and among species within ocean basins within one tissue (skin, the tissue with most estimates) (B, D). Figure S3. Scatterplot of 91 means from values of δ13C and adjusted values of δ15N [adjusted using baseline phytoplankton data extracted from Pethybridge et al., 2018, see Table S6] in adults of six marine turtle species. [file BRV-94-1947-s001.pdf]

SUPPORTING INFORMATION

Contents

Table S1. A survey of studies documenting regional spatial overlap of marine turtle species. *indicates only occasional sightings of a given species.

Fig. S1. High-resolution version of the images shown in Fig. 3.

Table S2. Summary table showing the number of studies using stable isotope analysis (SIA) of $\delta^{13}C$ and $\delta^{15}N$ to investigate the trophic ecology of marine turtles, organised by species and ocean basin.

Table S3. Summary table showing the number of studies using stable isotope analysis (SIA) of $\delta^{13}C$ and $\delta^{15}N$ to investigate the trophic ecology of marine turtles, organised by species and broader study topic introduced in the conceptual model shown in Fig. 1.

Table S4. Nested analyses of variance (ANOVAs) modelling interspecific differences in stable isotope values taking into account variation among sampled tissues and ocean basins.

Fig. S2. Exploratory data analyses comparing values of $\delta^{13}C$ and $\delta^{15}N$ among species within tissues within one ocean basin (Atlantic, the basin with most estimates) (A, C) and among species within ocean basins within one tissue (skin, the tissue with most estimates) (B, D).

Table S5. Nested analyses of variance (ANOVAs) modelling difference in stable isotope values among basins and tissues taking into account variation among species.

Table S6. Summary statistics of unadjusted $\delta^{15}N$ and adjusted $\delta^{15}N$ values from 91 data points of adult marine turtles used in our meta-analysis.

Fig. S3. Scatterplot of 91 means from values of $\delta^{13}C$ and adjusted values of $\delta^{15}N$ [adjusted using baseline phytoplankton data extracted from Pethybridge *et al.* (2018), see Table S6] in adults of six marine turtle species.

Table S1. A survey of studies documenting regional spatial overlap of marine turtle species. *indicates only occasional sightings of a given species.

Ocean Basin	Region	Cc	Cm	Dc	Ei	Nd	Lk	Lo	Reference
Atlantic Ocean	Eastern Atlantic (Europe)	■	■	■	■		■		Brongersma (1972, 1995)
	Eastern Atlantic (West Africa)	■	■	■	■			■	Brongersma (1972, 1995)
	Eastern Atlantic (Azores, Canary Islands, Madeira)	■	■	■	■		■		Brongersma (1972, 1995)
	Gulf of Mexico	■	■	■	■		■		Hildebrand (1995)
	Mediterranean	■	■	■	■			(*)	Geldiay *et al*. (1995); Sella (1995)
	Western Atlantic (South America)	(*)	■	■	■			■	Schulz (1995)
Pacific Ocean	Eastern Pacific (Central America)		■	■	■			■	Cornelius (1995)
	Eastern Pacific (Mexico)	■	■	■	■			■	Cliffton *et al*. (1995)
	Central Eastern Pacific (Colombia, Ecuador)	■	■	■	■			■	Green & Ortiz-Crespo (1995)
	Southeastern Pacific (Peru)	■	■	■	■			■	Hays Brown & Brown (1995)
	South Pacific (Fiji)	■	■	■	■				Pritchard (1995)
	South Pacific (New Zealand)	■	■	■	■			■	Pritchard (1995)
	Western Pacific (Japan)	■	■	■	■			■	Uchida & Nishiwaki (1995)
	Western Pacific (China Seas)	■	■	■	■				De Silva (1995); Huang (1995)
	Western Pacific (Philippines)		■	■	■			■	Carrascal de Celis (1995)
	Western Pacific (Indonesia & Thailand)	■	■	■	■			■	Polunin & Sumertha Nuitja (1995); Sutanto Suwelo *et al*. (1995)
	Western Pacific (Malaysia)		■	■	■			■	De Silva (1995); Kuan Tow & Moll (1995)
	Western Pacific (Papua New Guinea)	■	■	■	■	■		■	Spring (1995)
	Western Pacific (Australia)	■	■	■	■	■		■	Limpus (1995)
	Central Pacific (Hawaiian Archipelago, Samoas, Cook Islands)	■	■	■	■			■	Balazs (1995)
	Central Pacific (Micronesia)		■	■	■			■	Pritchard (1995)
Indian Ocean	Eastern Indian Ocean	■	■	■	■			■	Kar & Bhaskar (1995)
	Western Indian Ocean (Central Africa, Seychelles)	■	■	■	■			■	Frazier (1995)
	Western Indian Ocean (Arabian Waters)	■	■	■	■			■	Ross & Barwani (1995)
	Western Indian Ocean (South Africa)	■	■	■	■			■	Hughes (1995)

Fig. S1. High-resolution versions of the images shown in Fig. 3. Three Illustrations are provided for each species: (*i*) lateral view of skull; (*ii*) dorsal view of inside of lower jaw and rhamphotheca; (*iii*) ventral view of inside of upper jaw and rhamphotheca). These artist's renderings (executed by Dawn Witherington) are based on museum specimens housed in the Chelonian Research Institute.

Caretta caretta (i)

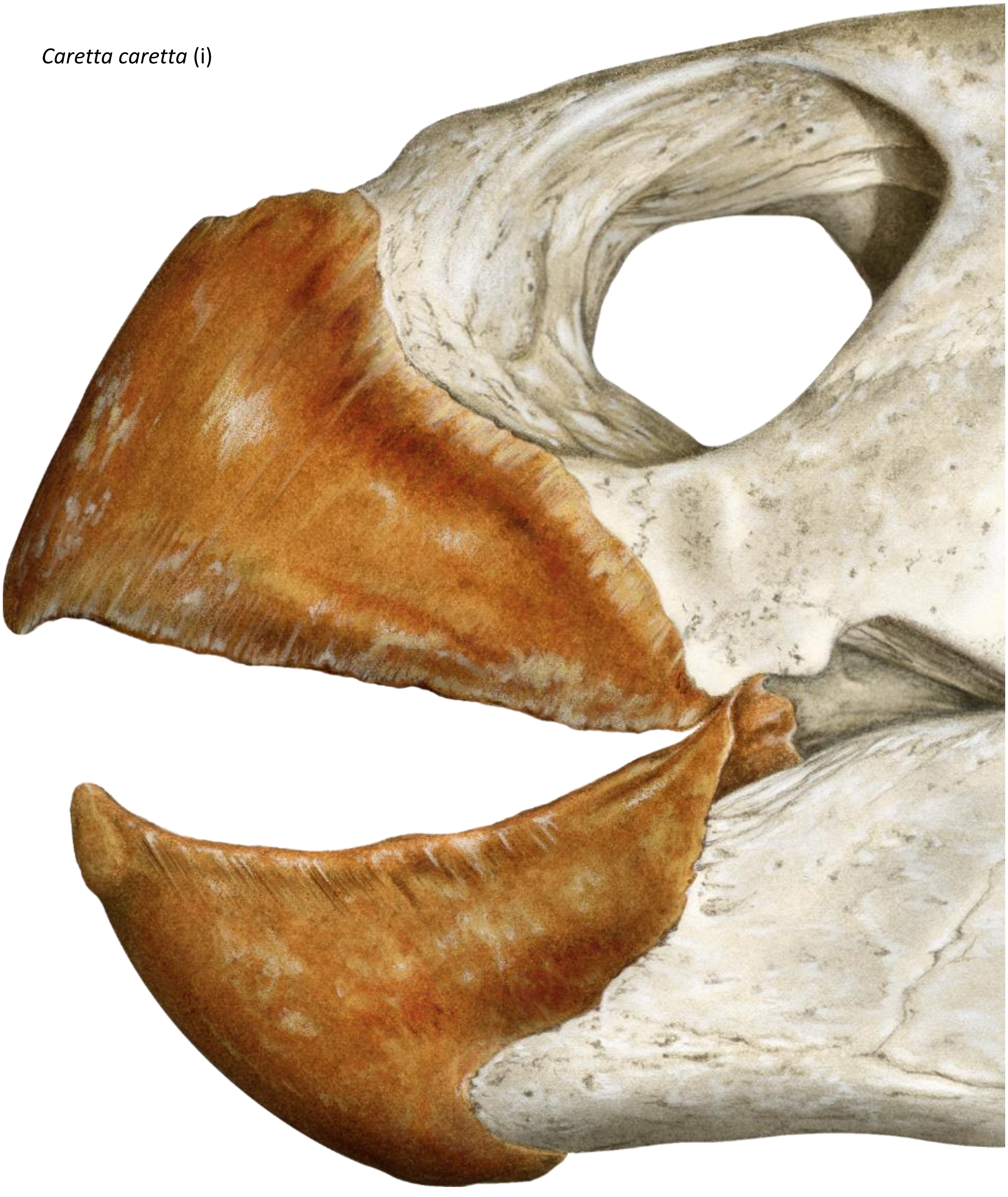

Caretta caretta (ii)

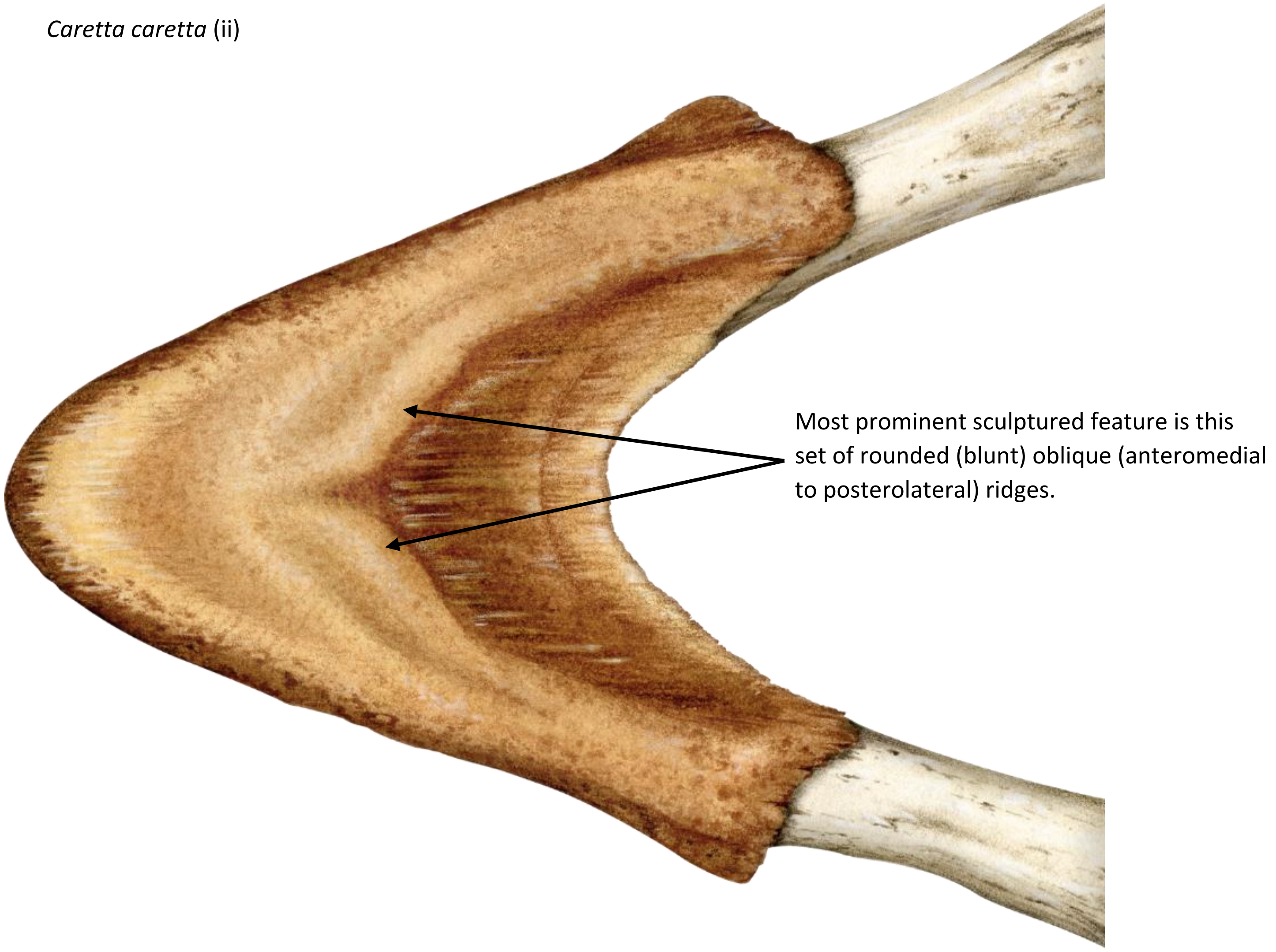

Caretta caretta (iii)

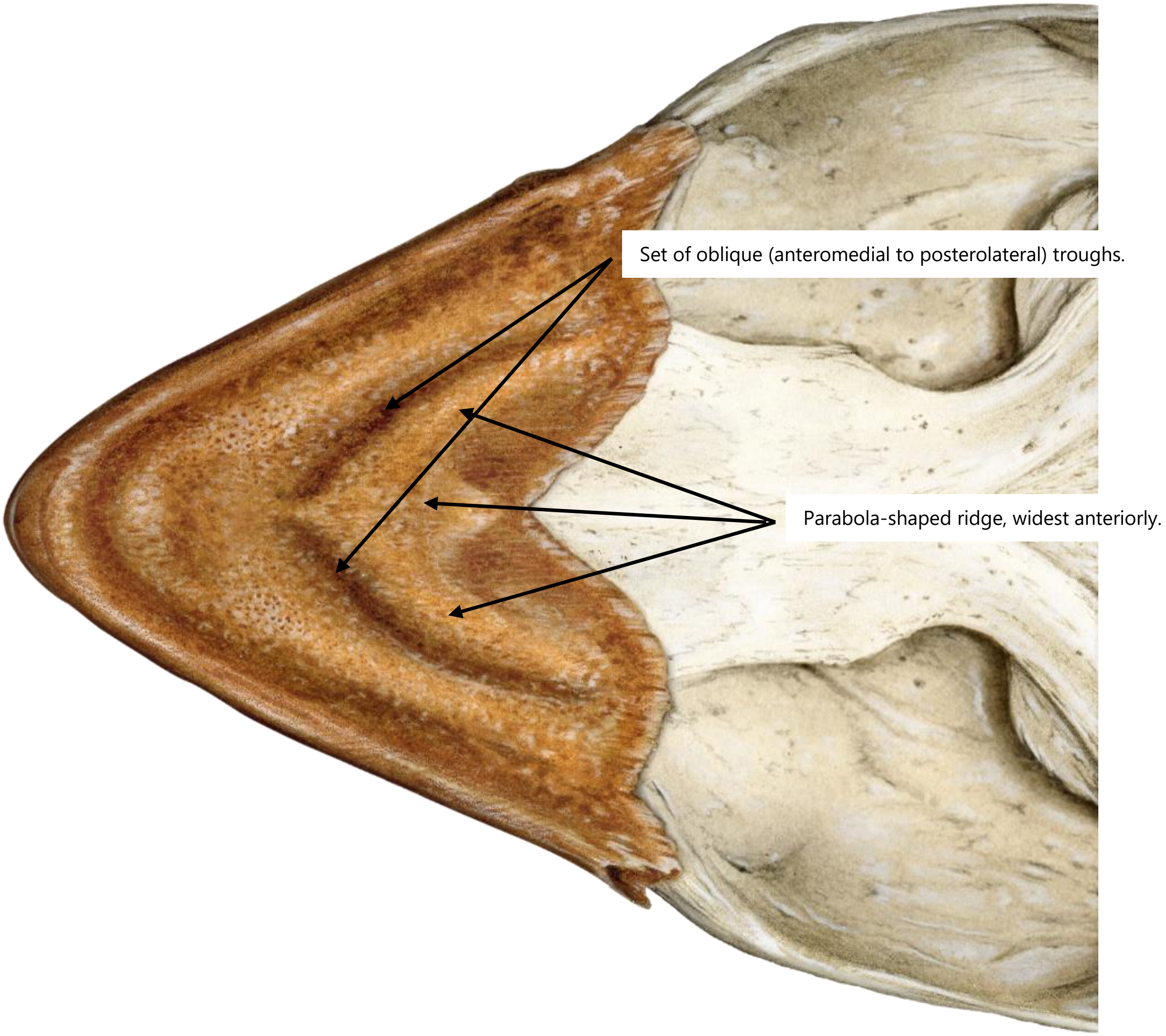

Chelonia mydas (i)

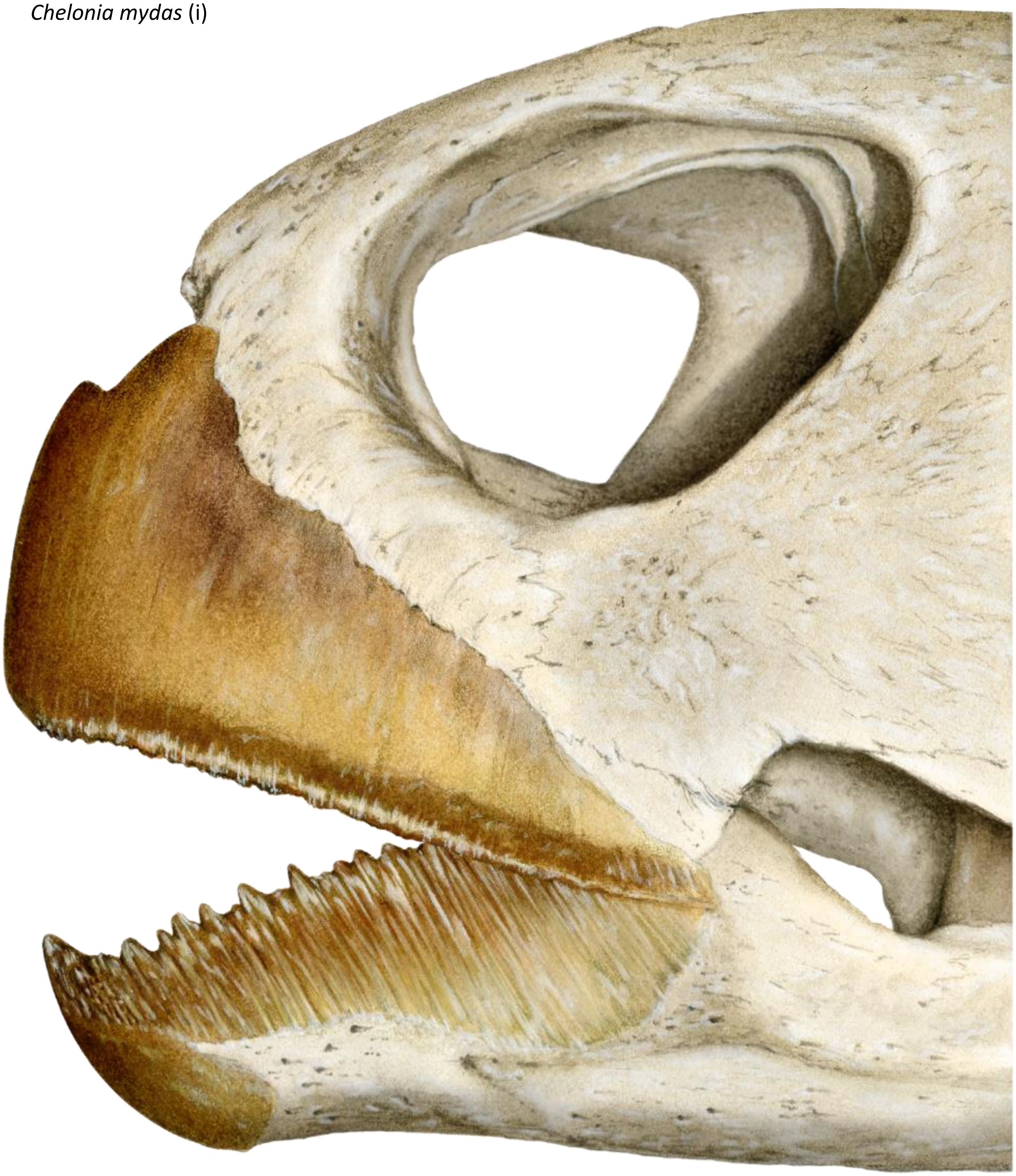

Chelonia mydas (ii)

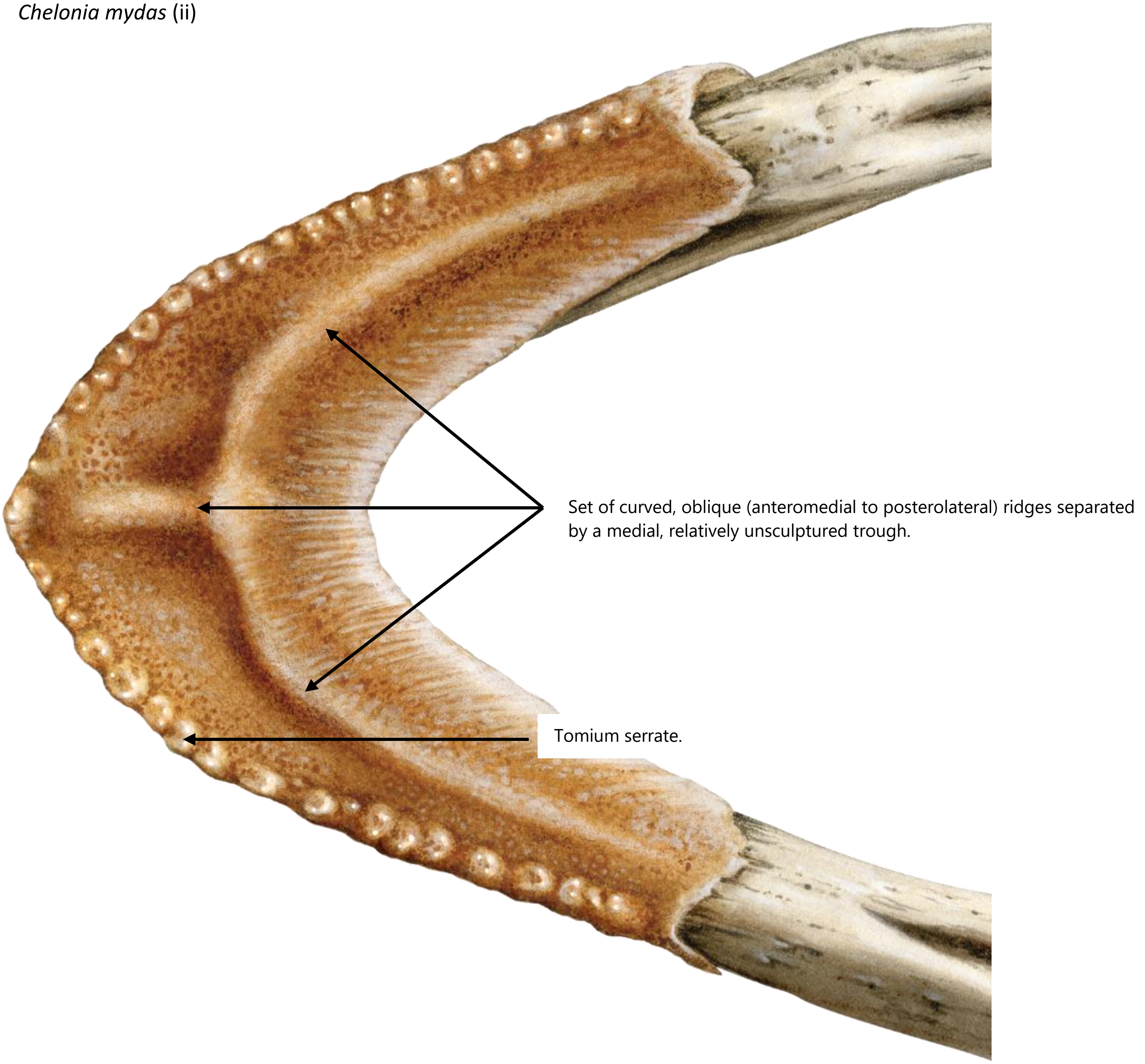

Chelonia mydas (iii)

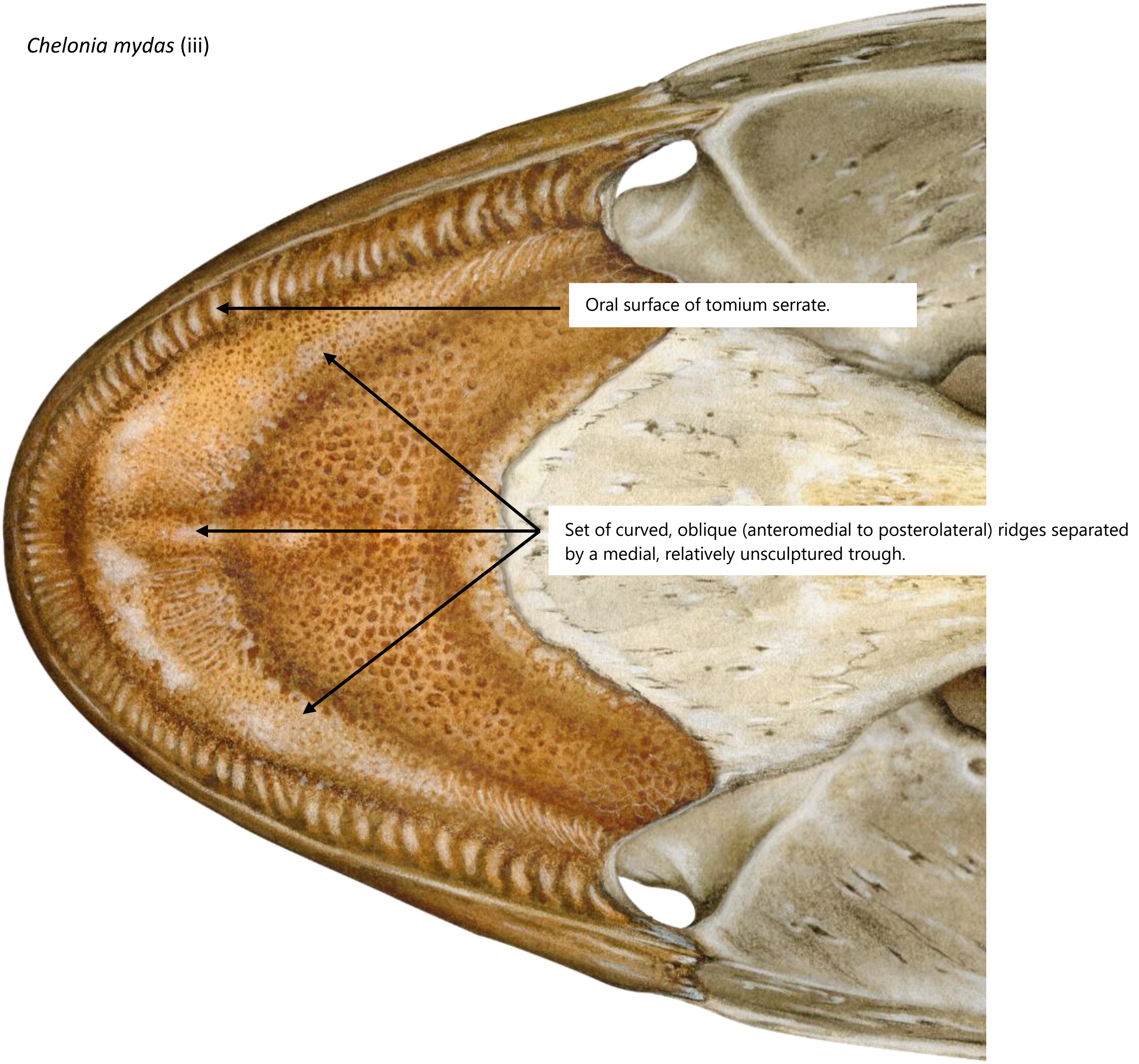

Eretmochelys imbricata (i)

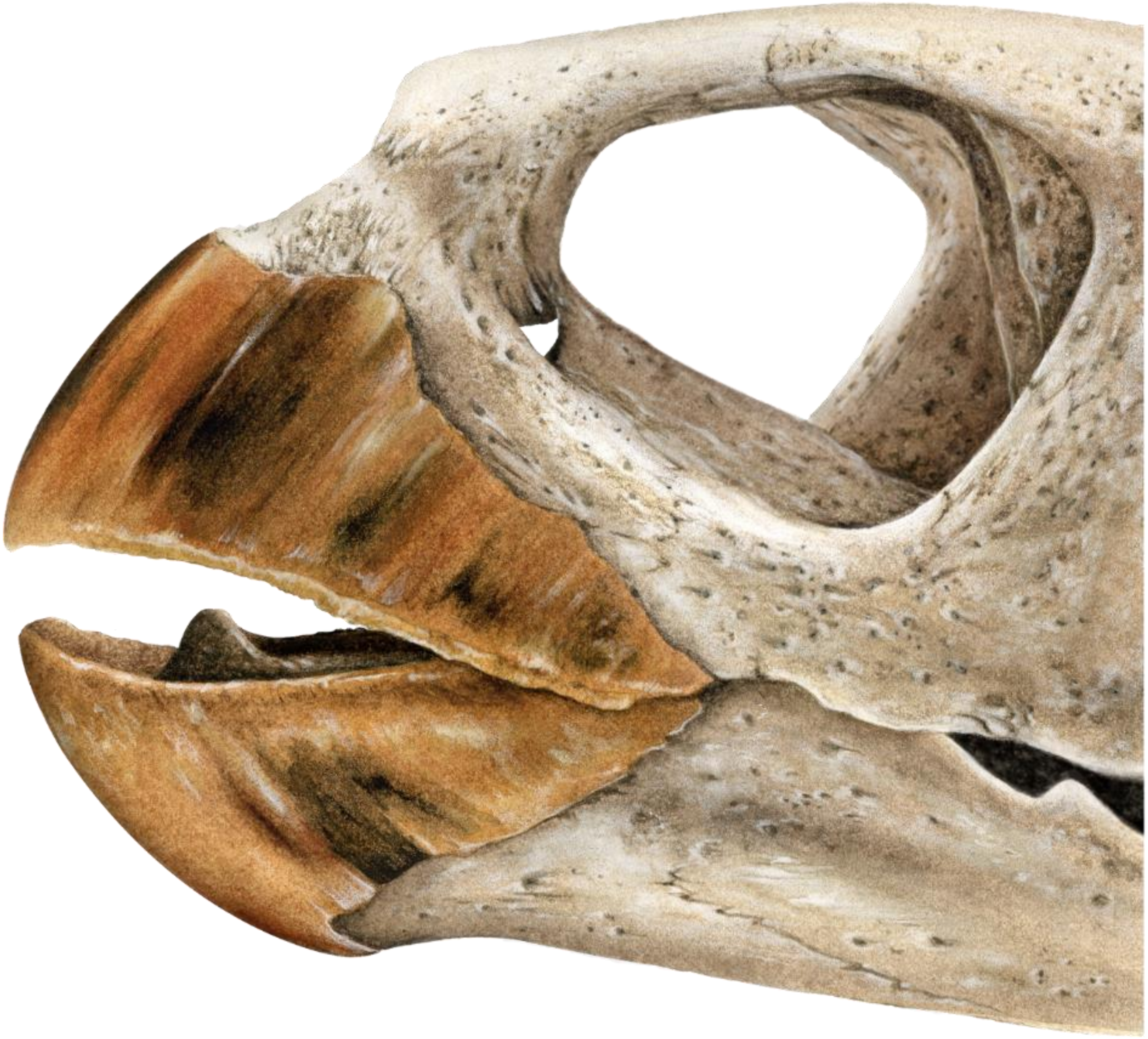

Eretmochelys imbricata (ii)

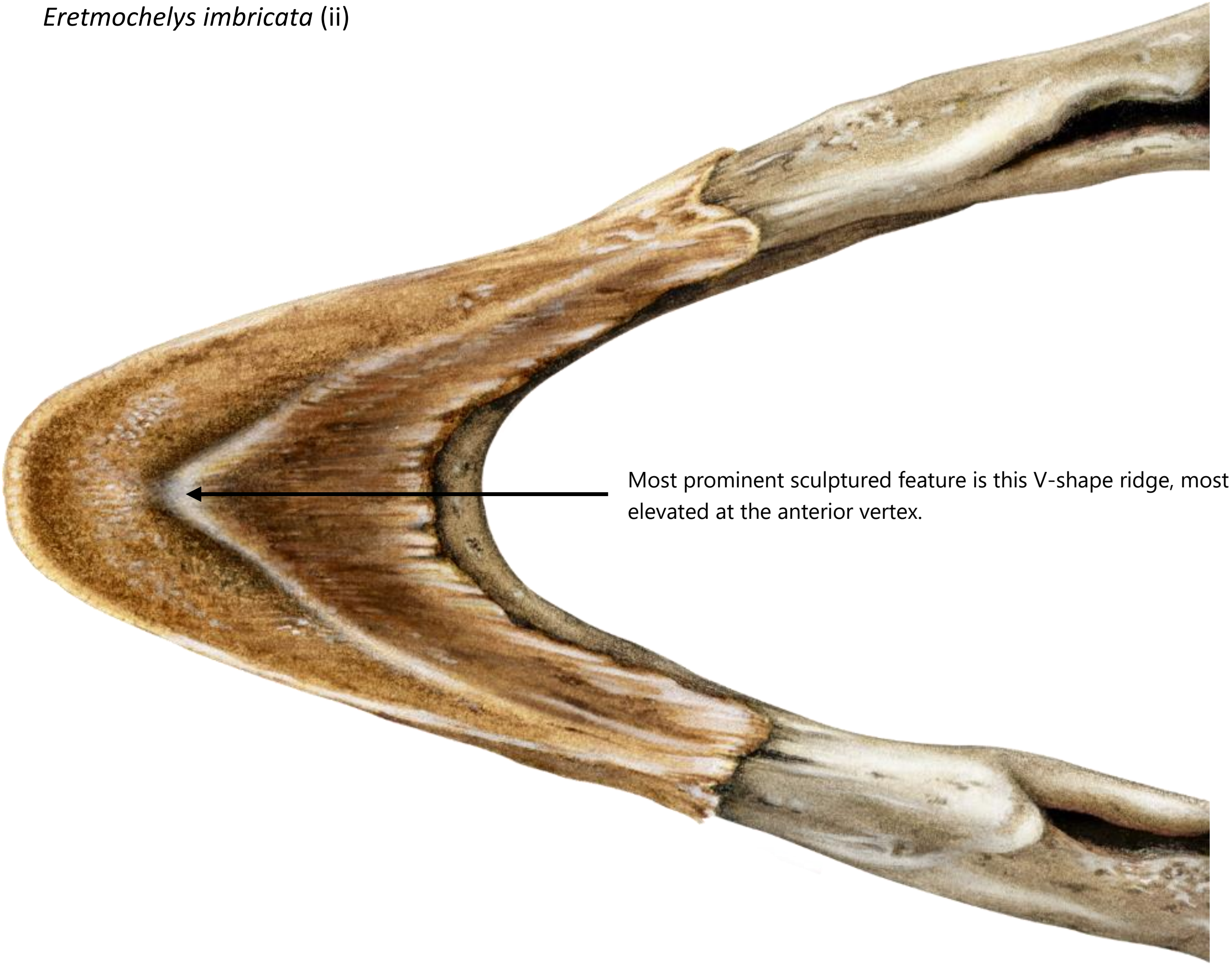

Eretmochelys imbricata (iii)

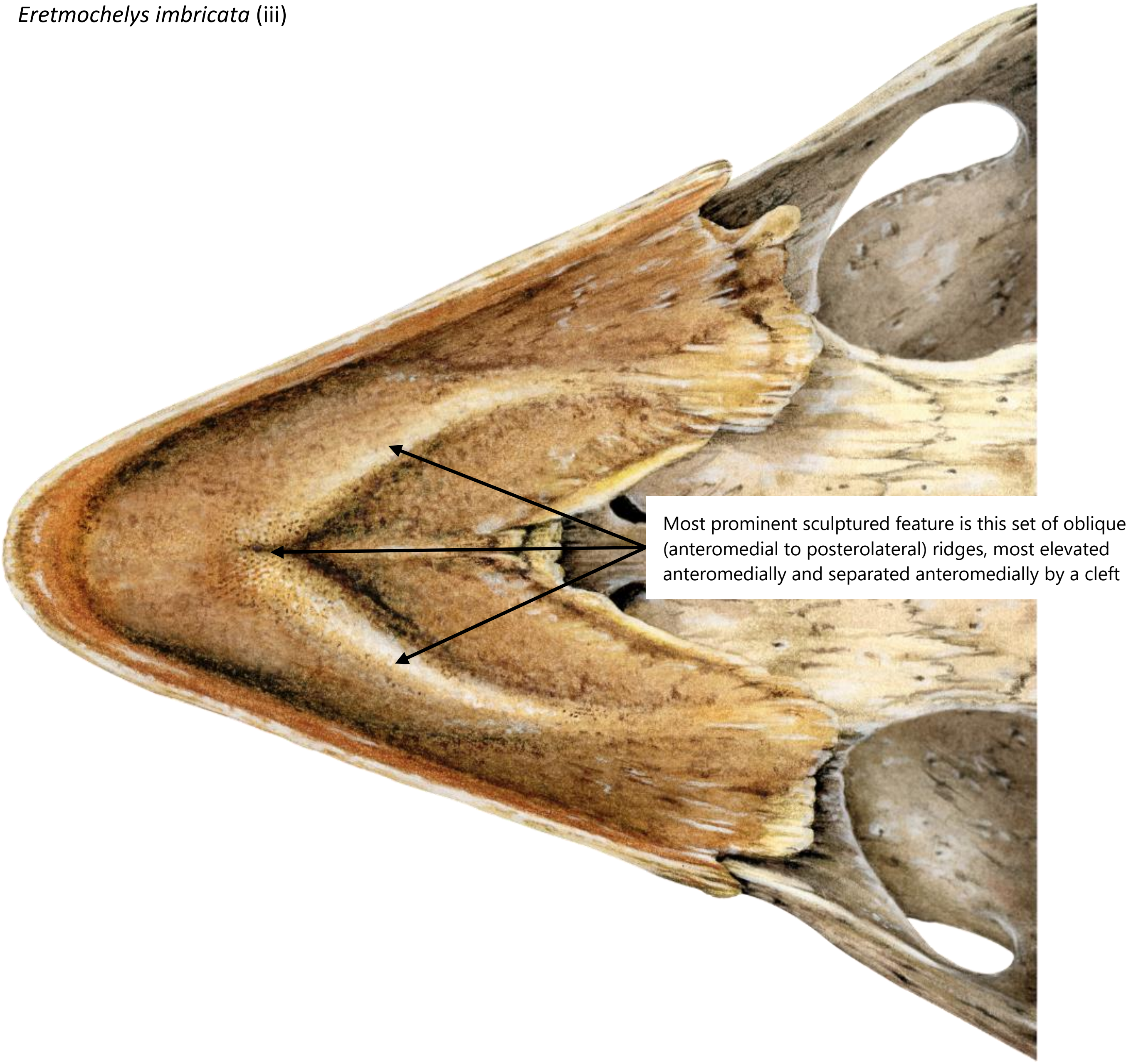

Lepidochelys kempii (i)

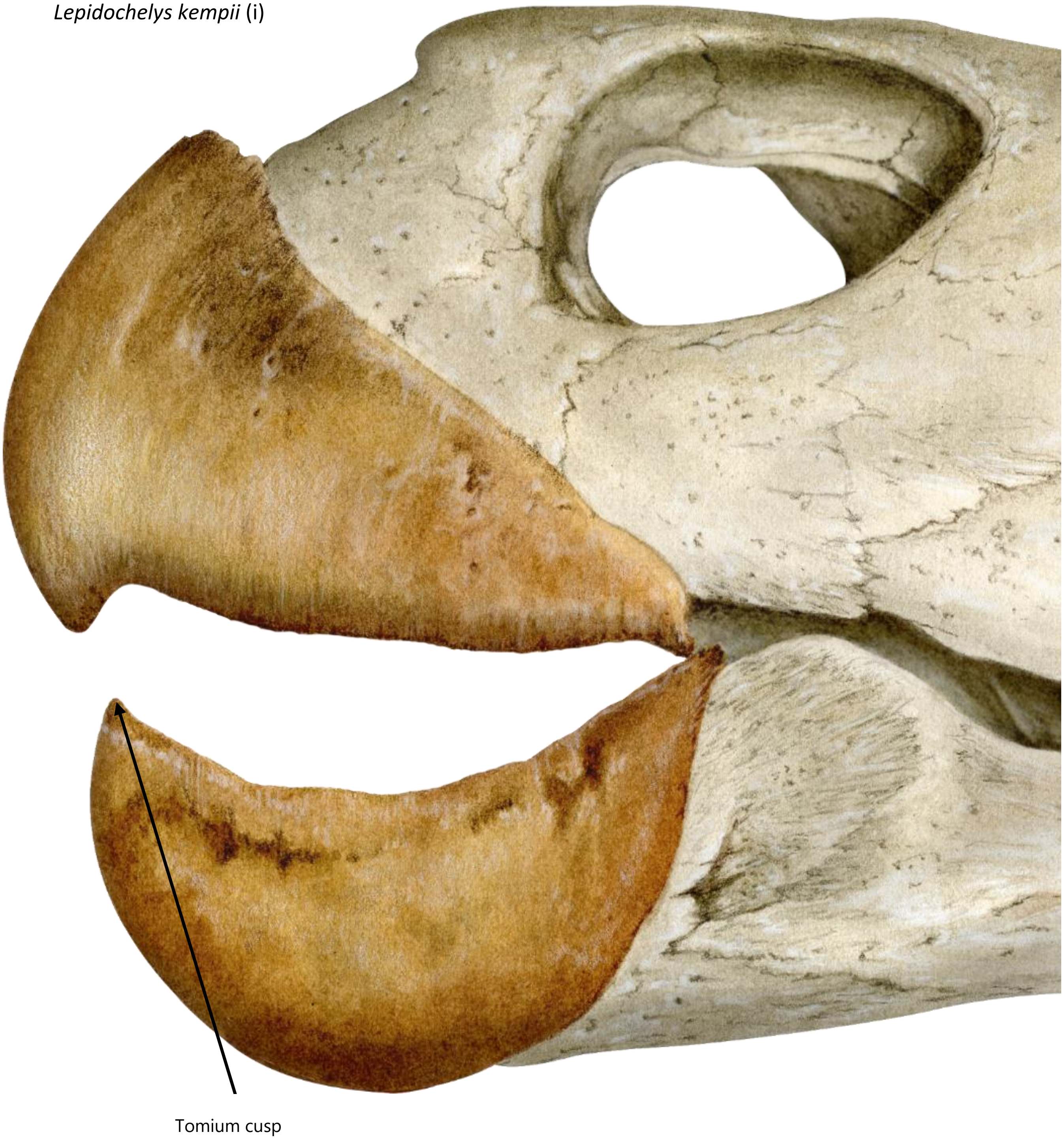

Lepidochelys kempii (ii)

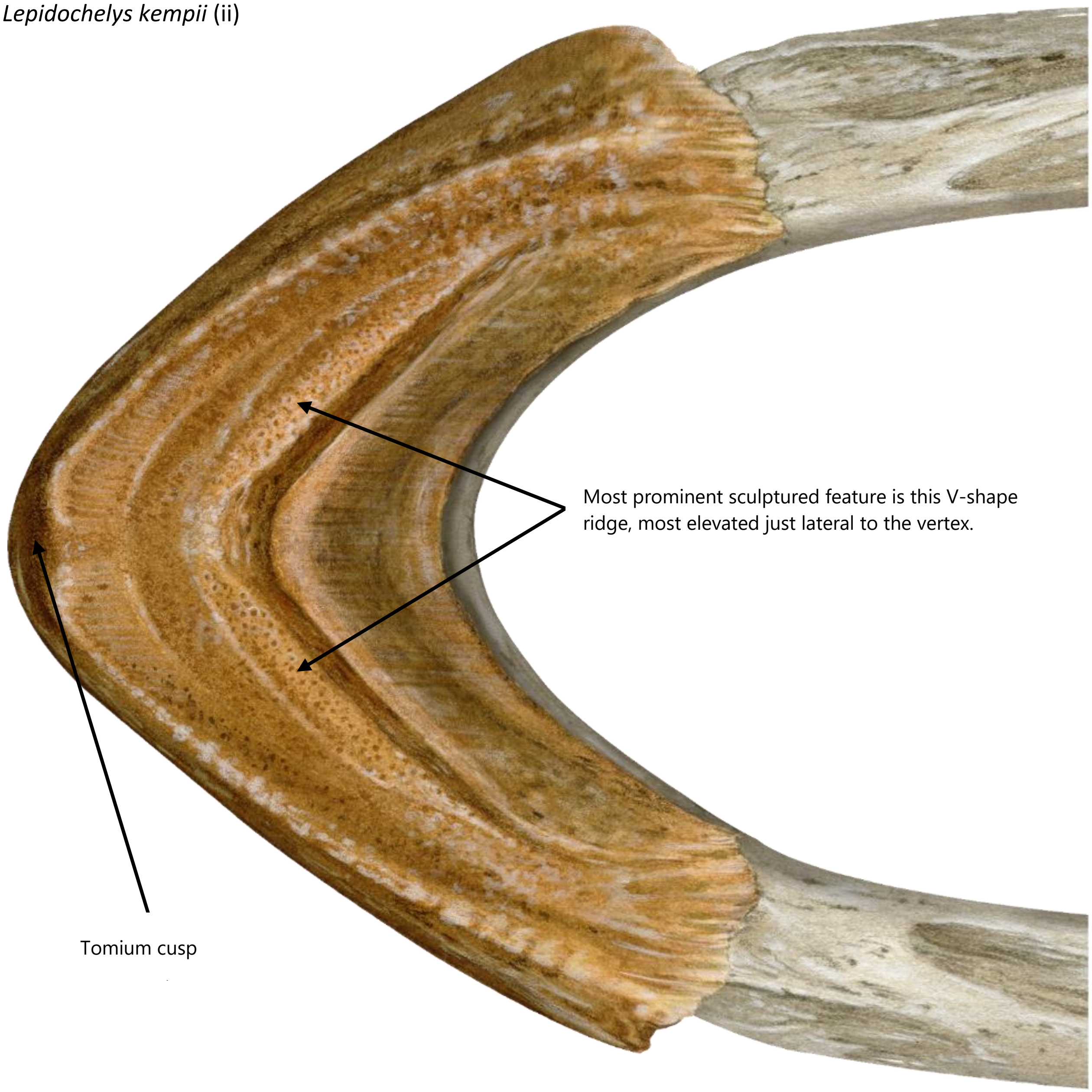

Lepidochelys kempii (iii)

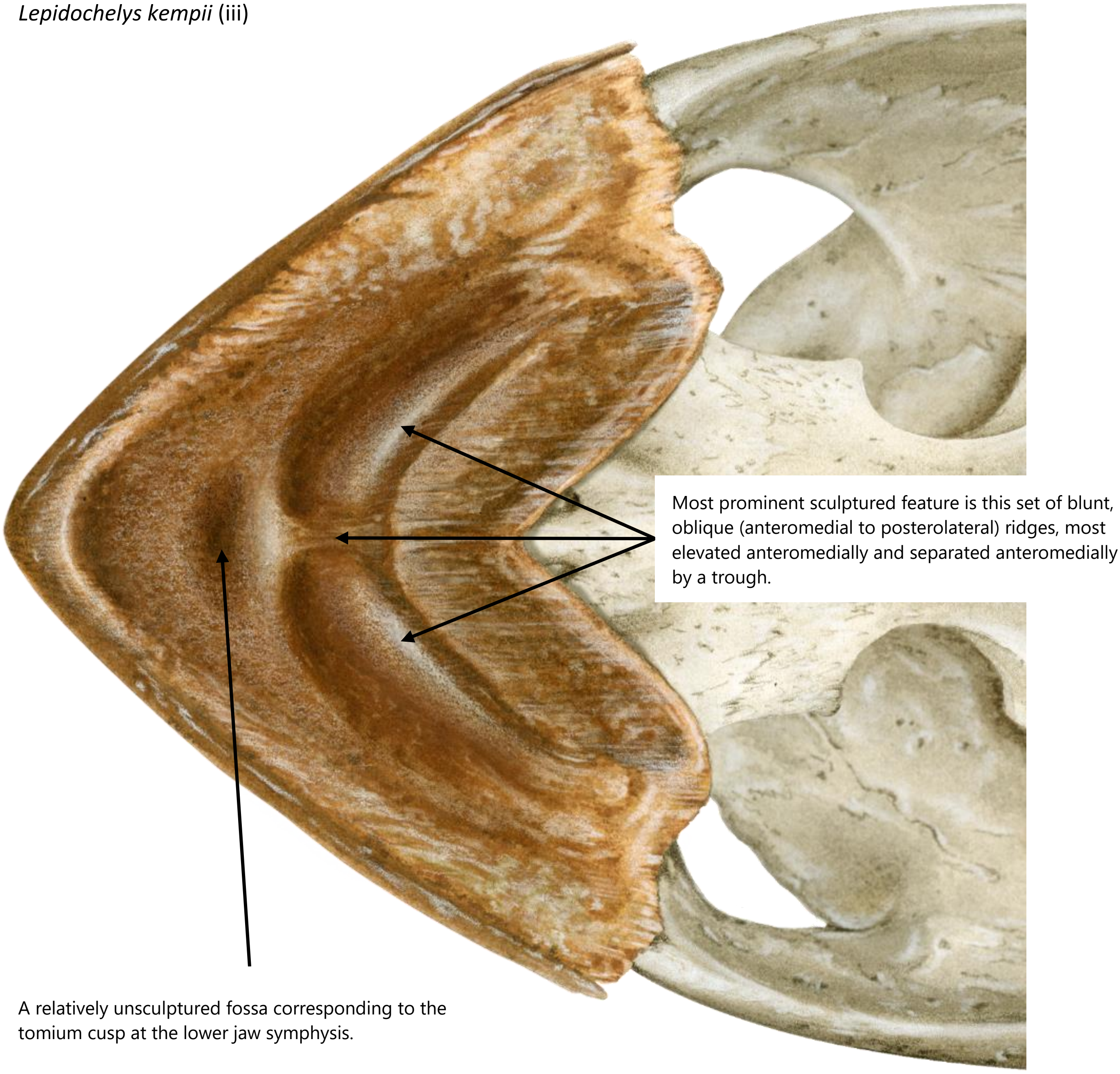

Lepidochelys olivacea (i)

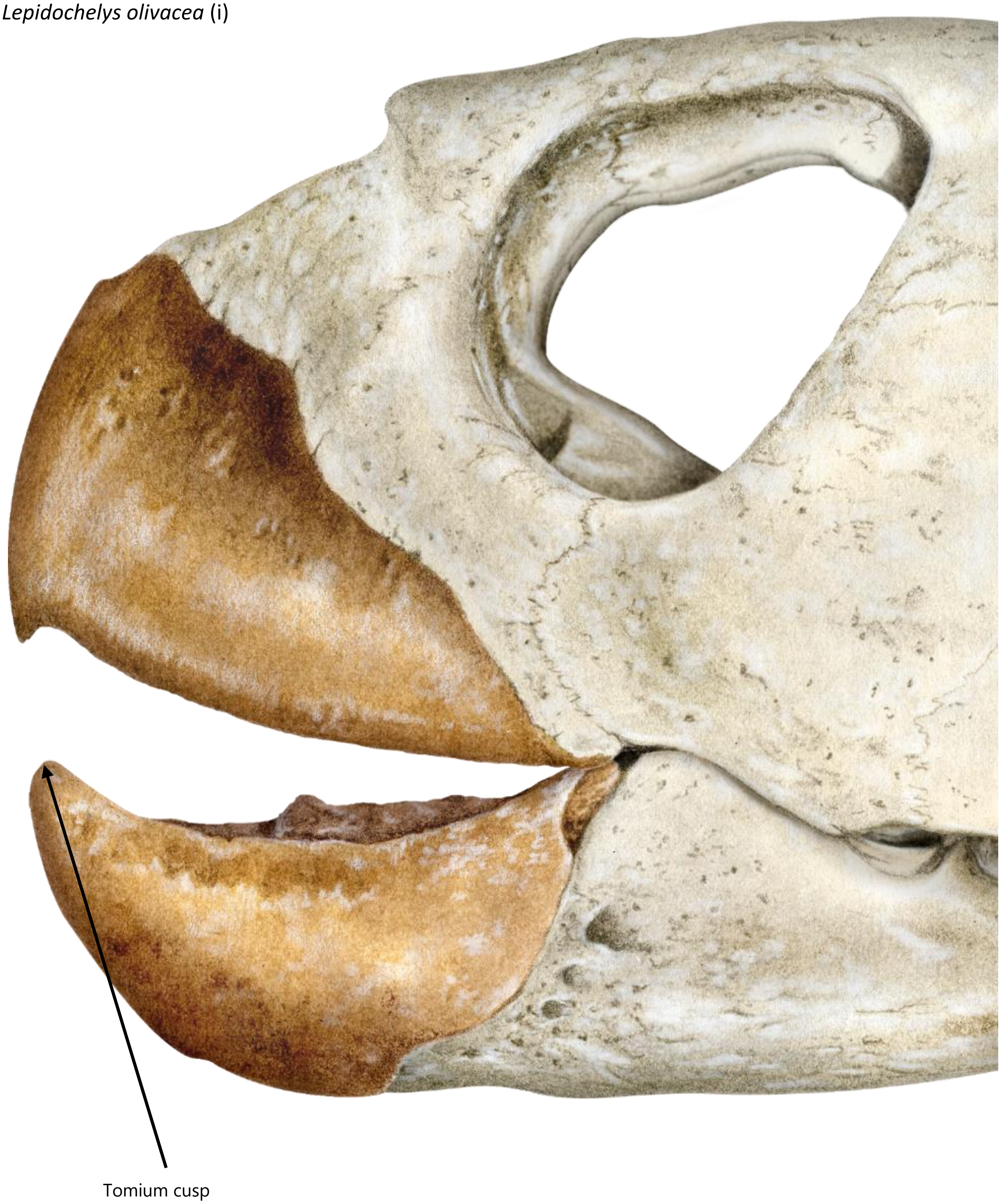

Lepidochelys olivacea (ii)

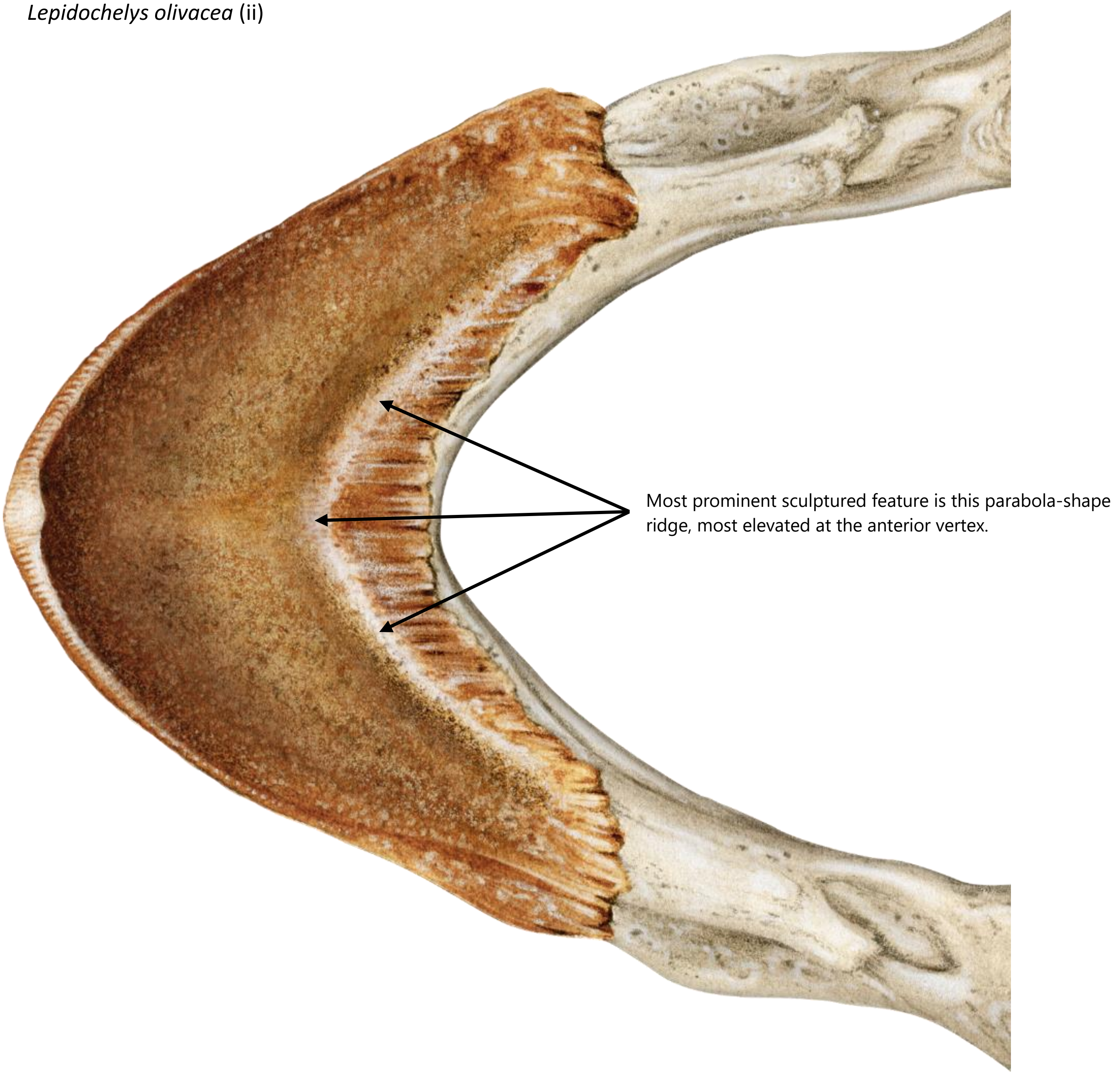

Lepidochelys olivacea (iii)

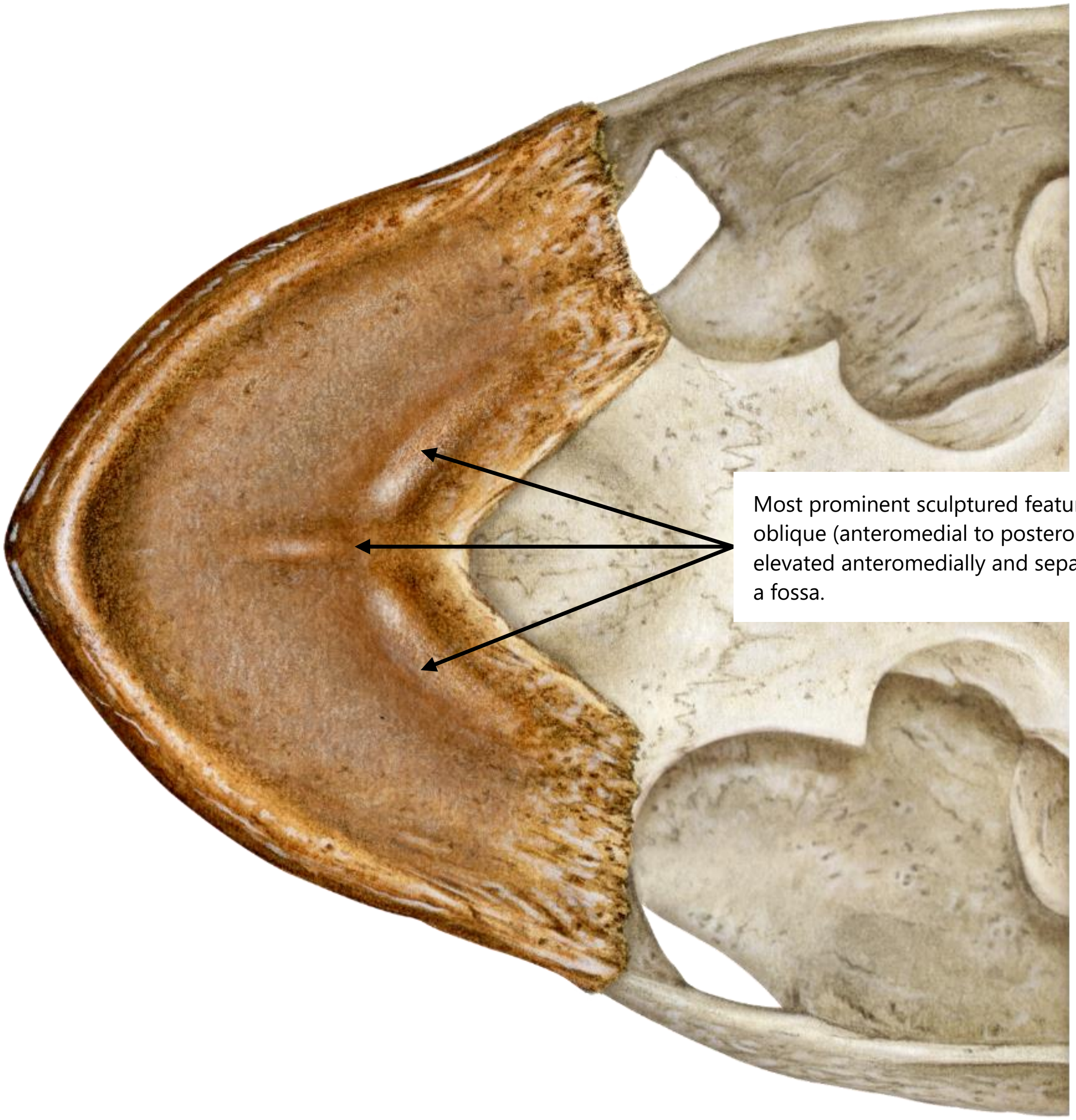

Natator depressus (i)

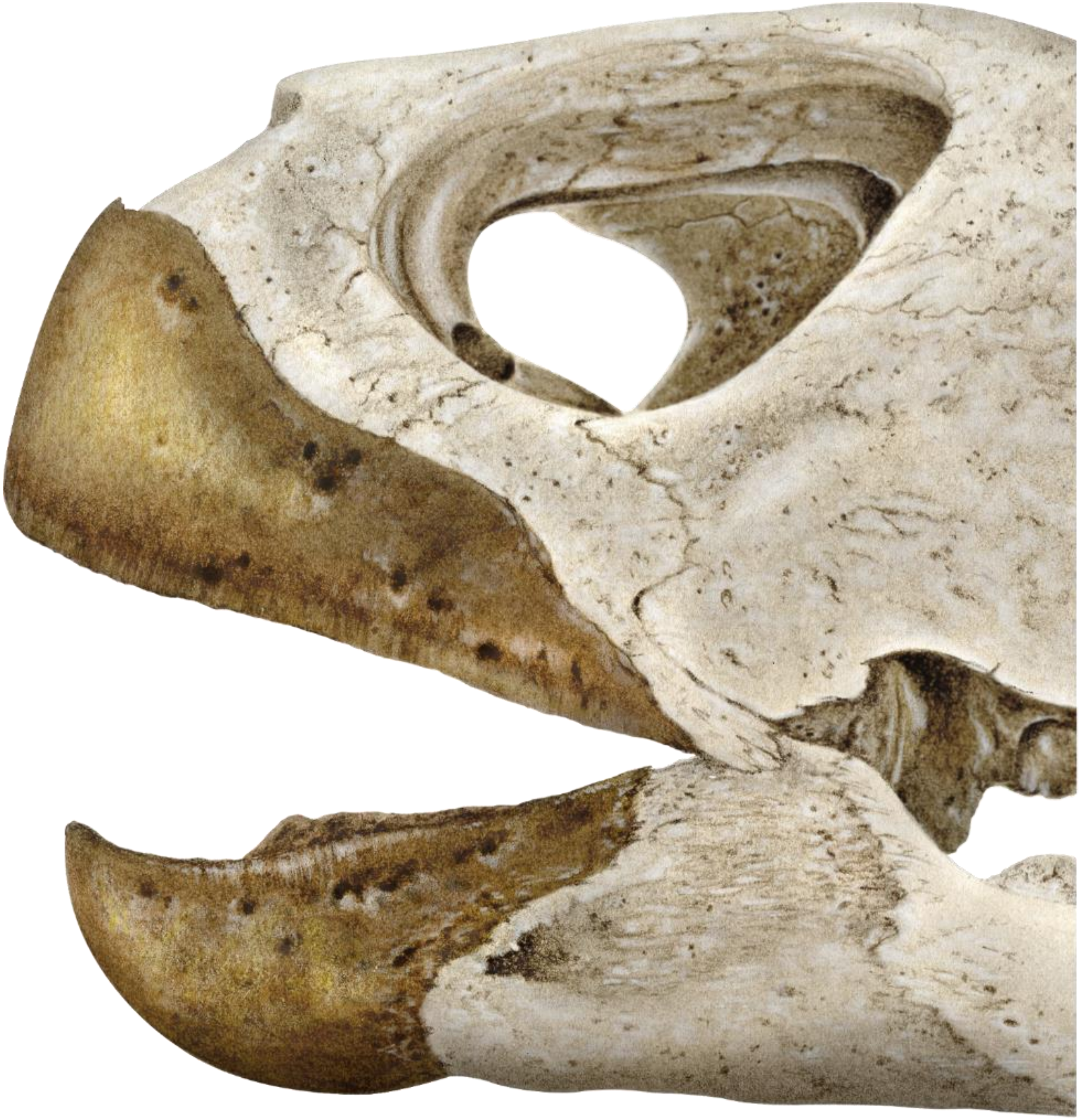

Natator depressus (ii)

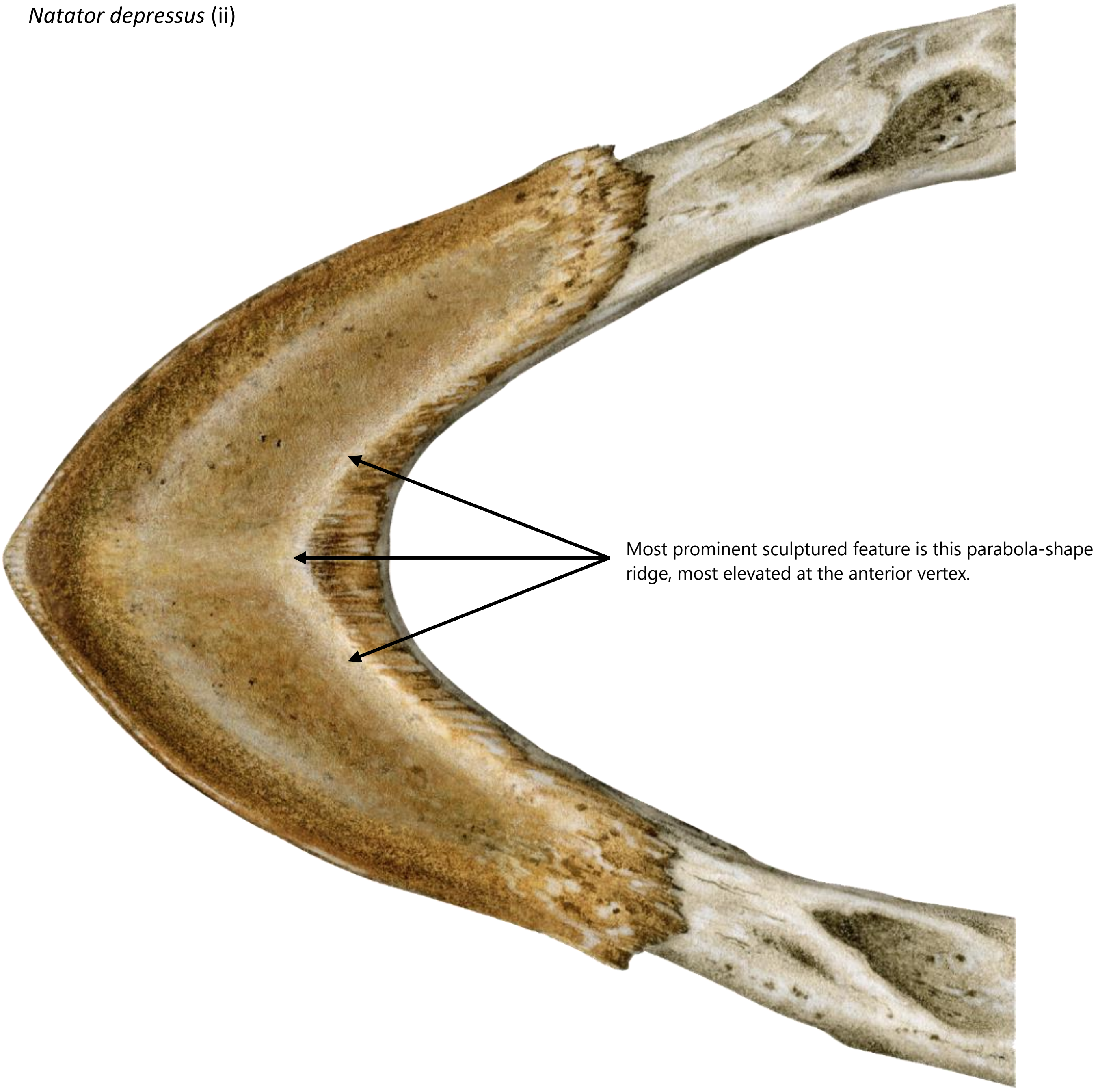

Natator depressus (iii)

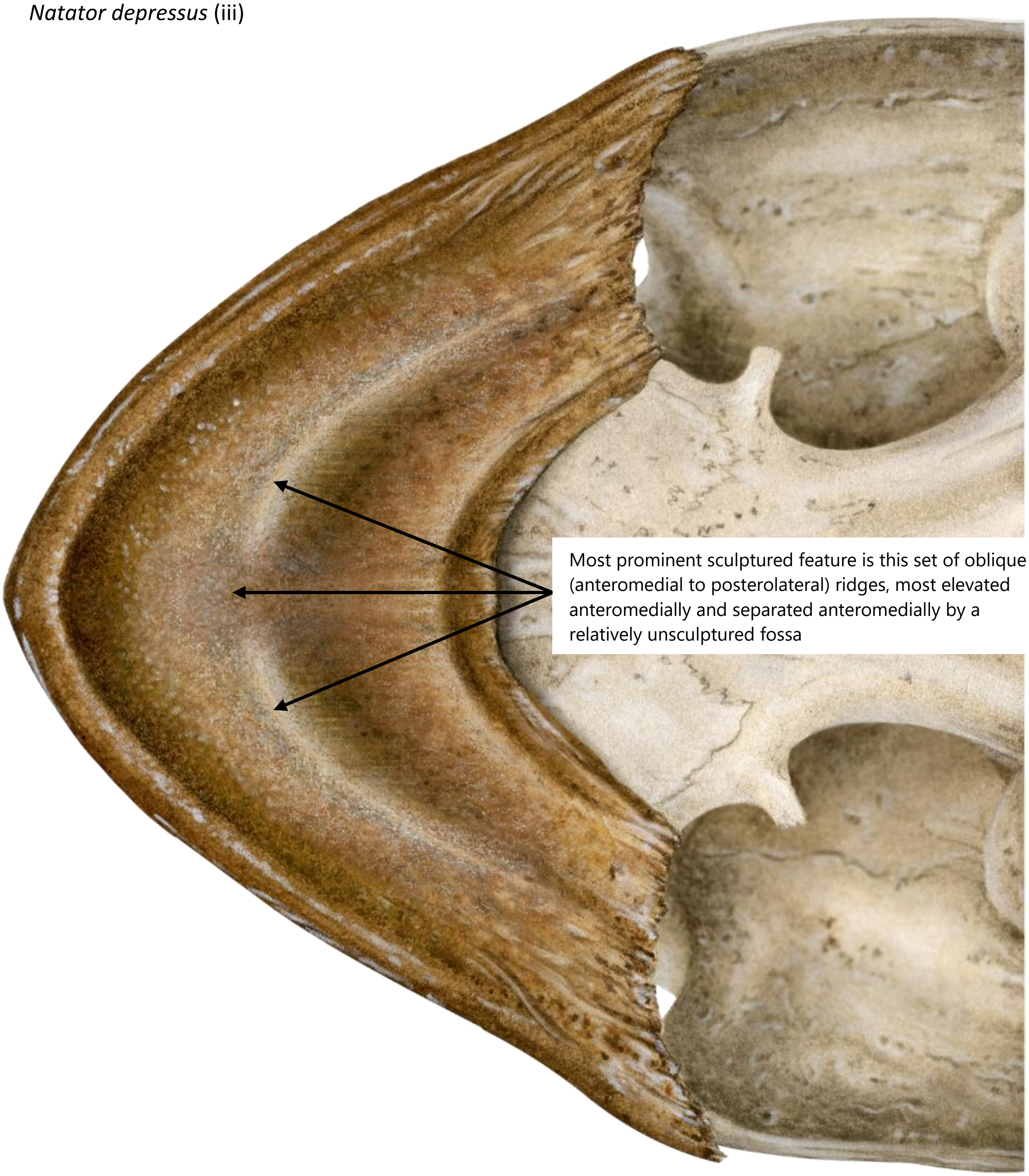

Dermochelys coriacea (i)

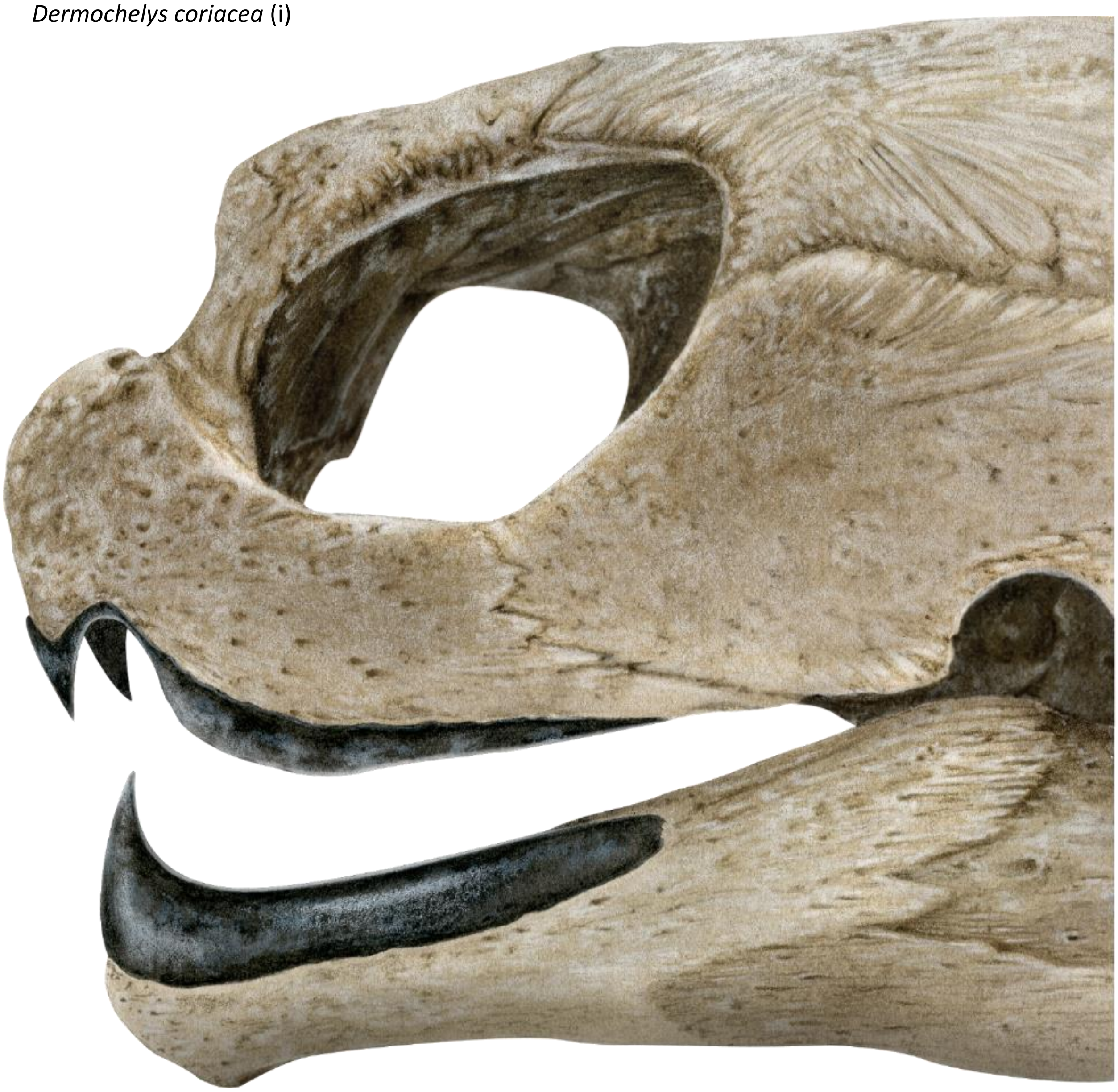

Dermochelys coriacea (ii)

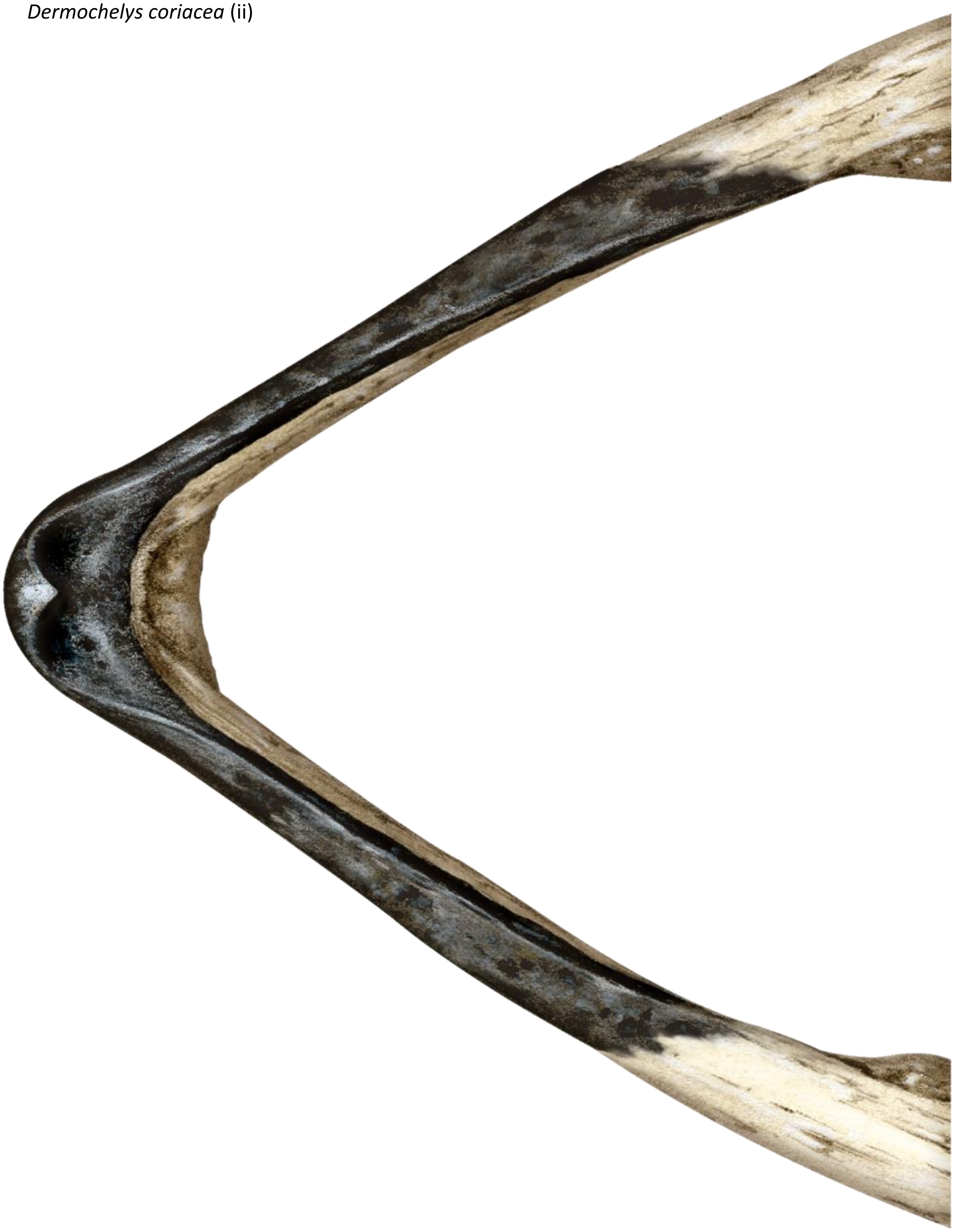

Dermochelys coriacea (iii)

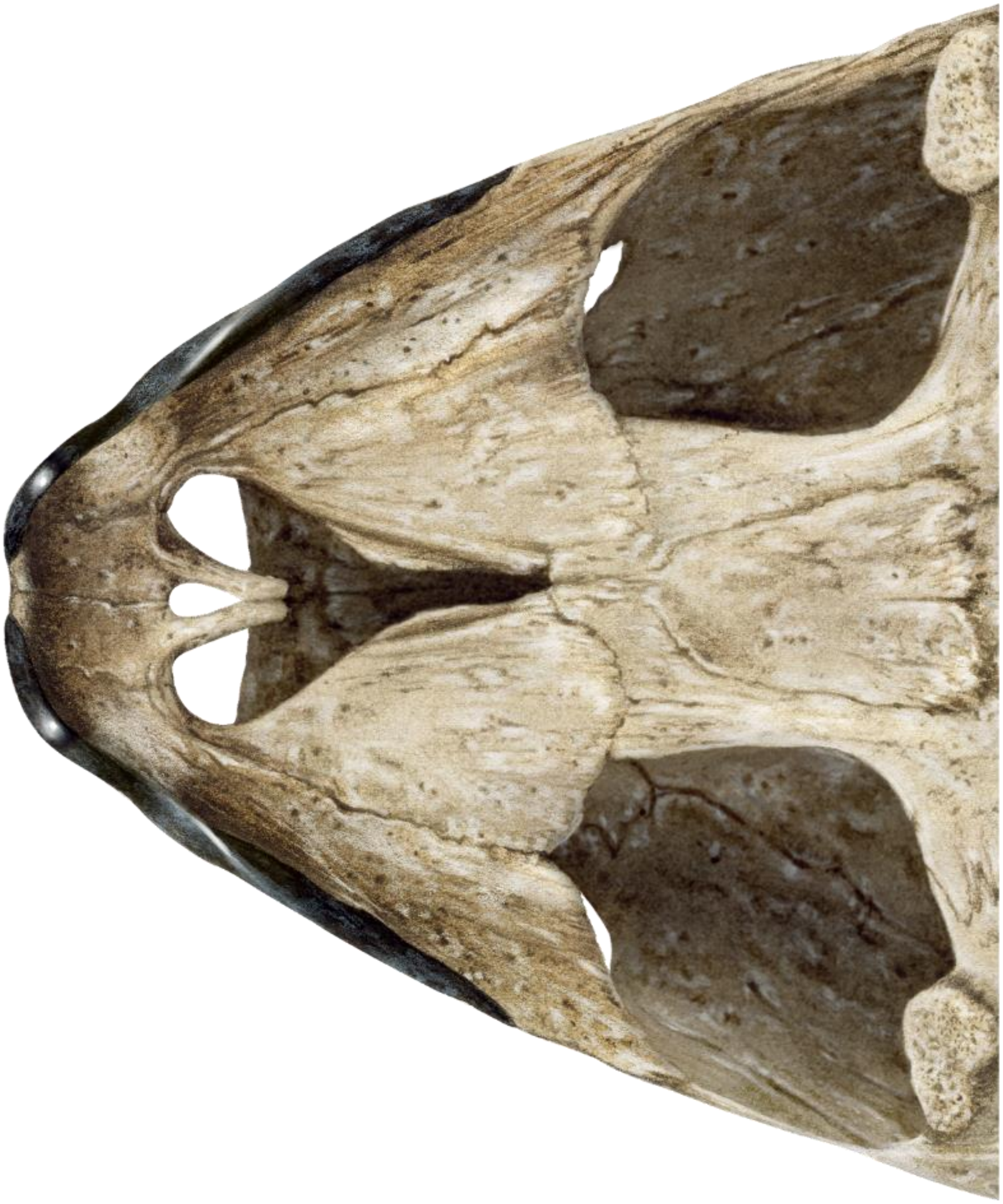

Table S2. Summary table showing the number of studies using stable isotope analysis (SIA) of $\delta^{13}C$ and $\delta^{15}N$ to investigate the trophic ecology of marine turtles, organised by species and ocean basin. N/A is a combination that is not possible (e.g. *Lepidochelys kempii* does not occur in the Pacific, Mediterranean, or Indian Ocean). NONE indicates this is a possible combination, but there are no estimates yet available. *Some studies involved more than one species or ocean basin. GoM, Gulf of Mexico.

		Marine turtle species						
		Caretta caretta	***Dermochelys coriacea***	***Chelonia mydas***	***Eretmochelys imbricata***	***Lepidochelys kempii***	***Lepidochelys olivacea***	***Natator depressus***
	**TOTAL**	56*	11*	45*	3*	2	7*	**NONE**
**Ocean basins**	**Atlantic Ocean (*inkl. GoM*)**	33	7	21	3	2	2	
	**Pacific Ocean**	14	3	16	1	*N/A*	5	
	**Mediterranean**	9	1	2	**NONE**	*N/A*	*N/A*	
	**Indian Ocean**	1	1	2	1	*N/A*	**NONE**	
	**Captivity**	1	1	3	**NONE**	**NONE**	**NONE**	

Table S3. Summary table showing the number of studies using stable isotope analysis (SIA) of $\delta^{13}C$ and $\delta^{15}N$ to investigate the trophic ecology of marine turtles, organised by species and broader study topic introduced in the conceptual model shown in Fig. 1. A, interspecific variation; B, inter-population variation; C, intra-population variation (C.1, ontogenetic variation; C.2, intersexual variation); D, inter-individual variation; M, method article. *Some studies involved more than one species or broader topic.

		**Marine turtle species**						
		Caretta caretta	***Dermochelys coriacea***	***Chelonia mydas***	***Eretmochelys imbricata***	***Lepidochelys kempii***	***Lepidochelys olivacea***	***Natator depressus***
**Broader study topic**	**TOTAL**	56*	11*	45*	3*	2*	7*	
**A**	7*	5	2	7	1	1	3	**NONE**
**B**	20	6	2	55	**NONE**	**NONE**	**NONE**	
**C.1**	46	16	1	29	2	1	2	
**C.2**	7	2	2	2	**NONE**	**NONE**	1	
**D**	41	25	6	6	1	1	3	
**M**	32	16	3	12	**NONE**	**NONE**	3	

Table S4. Nested analyses of variance (ANOVAs) modelling interspecific differences in stable isotope values taking into account variation among sampled tissues and ocean basins. The least-squares means and standard errors derived from these analyses are plotted in Fig. S2. (a) Within-basin (Atlantic) analysis of $\delta^{13}C$ values, nesting species within tissues; (b) within-tissue (skin) analysis of $\delta^{13}C$ values, nesting species within basins; (c) within-basin (Atlantic) analysis of $\delta^{15}N$ values, nesting species within tissues; (d) within-tissue (skin) analysis of $\delta^{15}N$ values, nesting species within basins.

**Source of variation**	**DF**	**type III SS**	***F*-ratio**	**prob>*F***
**(a) $\delta^{13}C$, nesting species within tissues** MODEL- adjusted R^2= 0.808107	20	578.83878	11.7387	<0.0001
Effects				
Tissue	6	269.98966	18.2511	<0.0001
Species[Tissue]	14	372.32957	10.7868	<0.0001
Error	31	76.43103		
corrected total	51	655.26981		
**(b) $\delta^{13}C$, nesting species within basins** MODEL- adjusted R^2= 0.688911	12	188.28612	6.5363	0.0002
Effects				
Basin	3	54.36560	7.5491	0.0018
Species[Basin]	9	135.70016	6.2811	0.0005
Error	18	43.20936		
corrected total	30	231.49548		
**(c) $\delta^{15}N$, nesting species within tissues** MODEL- adjusted R^2= 0.484669	19	168.69364	3.4255	0.0013
Effects				
Tissue	6	75.685619	4.8668	0.0014
Species[Tissue]	13	78.858091	2.3404	0.0269
Error	30	76.43103		
corrected total	49	246.45120		
**(d) $\delta^{15}N$, nesting species within basins** MODEL- adjusted R^2= 0.224991	12	100.33152	1.7258	0.1432
Effects				
Basin	3	36.692136	2.5245	0.0901
Species[Basin]	9	59.469103	1.3639	0.2742
Error	18	87.20590		
corrected total	30	187.53742		

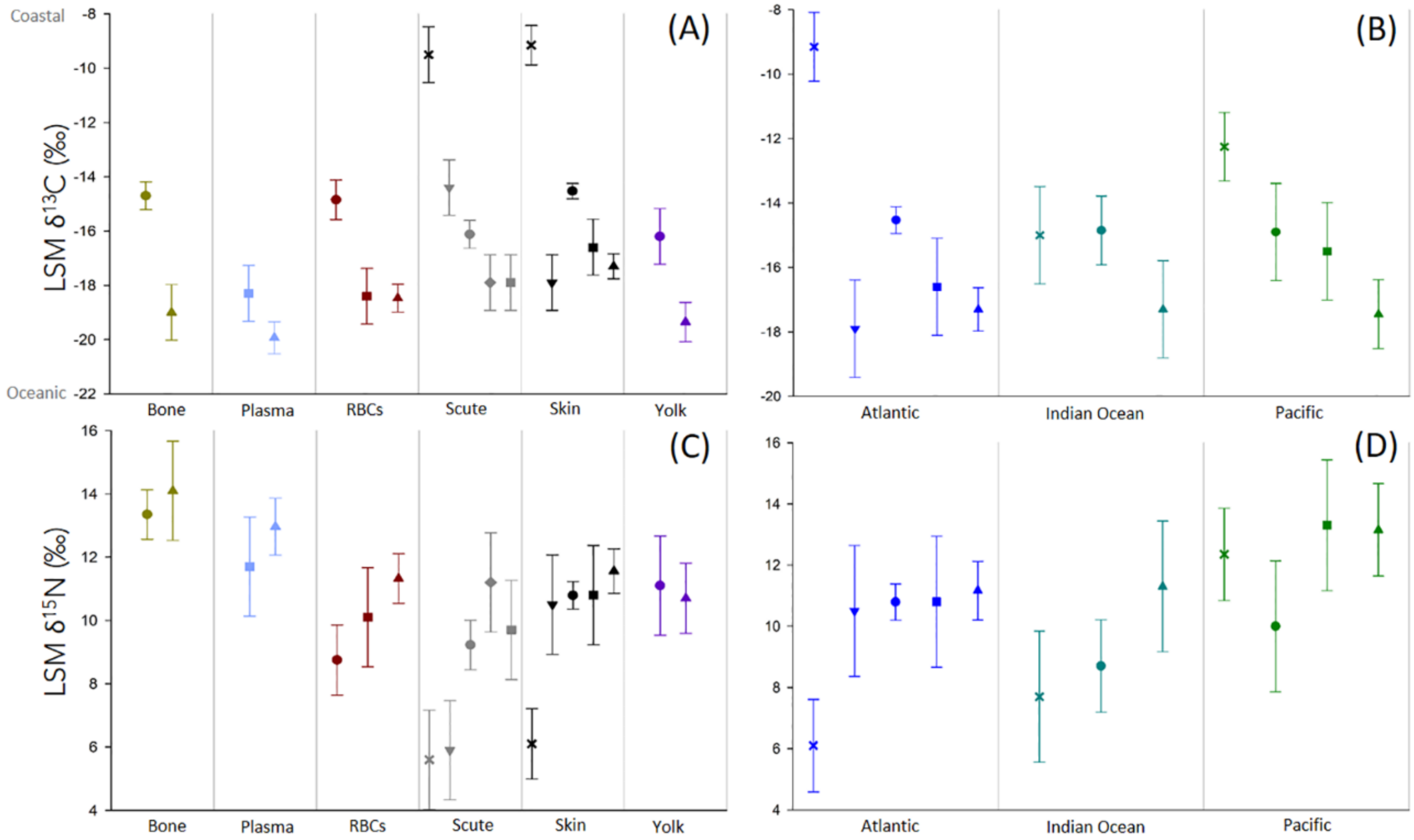

Fig. S2. Exploratory data analyses comparing values of $\delta^{13}C$ and $\delta^{15}N$ among species within tissues within one ocean basin (Atlantic, the basin with most estimates) (A, C) and among species within ocean basins within one tissue (skin, the tissue with most estimates) (B, D). Plotted values are least-squares means (LSMs) and standard errors for six different marine species (*C. caretta*, circle; *C. mydas*, cross; *D. coriacea*, triangle; *E. imbricata*, inverted triangle; *L. kempii*, diamond; *L. olivacea*, square) derived from separate nested ANOVAs for each isotope ($\delta^{13}C$, $\delta^{15}N$) within the Atlantic (Table S4a, c) and skin (Table S4b,d), respectively.

Table S5. Nested analyses of variance (ANOVAs) modelling difference in stable isotope values among basins and tissues taking into account variation among species. (a) Within-basin (Atlantic) analysis of $\delta^{13}C$ values, nesting tissues within species; (b) within-basin (Atlantic) analysis of $\delta^{15}N$ values, nesting tissues within species; (c) within-tissue (skin) analysis of $\delta^{13}C$ values, nesting basins within species; (d) within-tissue (skin) analysis of $\delta^{15}N$ values, nesting basins within species.

**Source of variation**	**DF**	**type III SS**	***F*-ratio**	**prob>*F***
**(a) $\delta^{13}C$, nesting tissue within species** MODEL- adjusted R^2= 0.852385	19	325.16861	15.8918	<0.0001
Effects				
Species	5	234.73048	43.5932	<0.0001
Tissue[Species]	14	30.56957	2.0276	0.0512
Error	30	32.30743		
corrected total	49	357.47605		
**(b) $\delta^{15}N$, nesting tissue within species** MODEL- adjusted R^2= 0.444961	19	152.97358	3.0675	0.0030
Effects				
Species	5	95.242100	7.2573	0.0001
Tissue[Species]	14	56.150633	1.5281	0.1606
Error	30	78.74124		
corrected total	49	231.71482		
**(c) $\delta^{13}C$, nesting basin within species** MODEL- adjusted R^2= 0.806887	12	211.62683	11.7940	<0.0001
Effects				
Species	4	141.90162	23.7246	<0.0001
Basin[Species]	8	33.53107	2.8030	0.0311
Error	19	28.41077		
corrected total	31	240.03760		
**(d) $\delta^{15}N$, nesting basin within species** MODEL- adjusted R^2= 0.452576	12	100.00242	3.1357	0.0128
Effects				
Species	4	70.081556	6.5926	0.0017
Basin[Species]	8	17.410822	0.8189	0.5957
Error	19	50.49431		
corrected total	31	150.49672		

Table S6. Summary statistics of unadjusted $\delta^{15}N$ and adjusted $\delta^{15}N$ values from 91 data points of adult marine turtles used in our meta-analysis. The adjustment was made using mean baseline phytoplankton $\delta^{15}N$ values for different ocean regions in four different basins (Atlantic, Pacific, Indian Ocean, Mediterranean) extracted from Pethybridge *et al.* (2018) with PlotDigitizer 2.6.8. The mean values were subtracted from raw $\delta^{15}N$ mean values of adult turtle populations to account for basin effects in the raw data. The ocean region in which each sampled marine turtle population is likely foraging was ball-parked. The baseline phytoplankton $\delta^{15}N$ values differed by less than one trophic step (range ~2–4‰) among most ocean regions, with the exception of the central trans-Pacific Ocean (~0‰) and the eastern Pacific Ocean (wide range in values and mean enriched in ^{15}N, ~7‰). CV, coefficient of variation.

			**$\delta^{15}N$ values**			**Adjusted $\delta^{15}N$ values**		
	**Species**	***N***	**CV**	**Range**		**CV**	**Range**	
				**Min.**	**Max.**		**Min.**	**Max.**
CHELONIIDAE	*Caretta caretta*	48	0.209	7.3	16.6	0.316	2.5	13.3
	Chelonia mydas	9	0.204	5.1	9.2	0.870	0.8	13.2
	Eretmochelys imbricata	4	0.226	5.9	10.5	0.508	1.1	5.9
	Lepidochelys kempii	1	NA	11.2	11.2	NA	6.4	6.4
	Lepidochelys olivacea	7	0.157	9.7	14.3	0.135	4.9	7
DERMOCHELYIDAE	*Dermochelys coriacea*	22	0.135	9.5	16.2	0.221	4.7	12.1

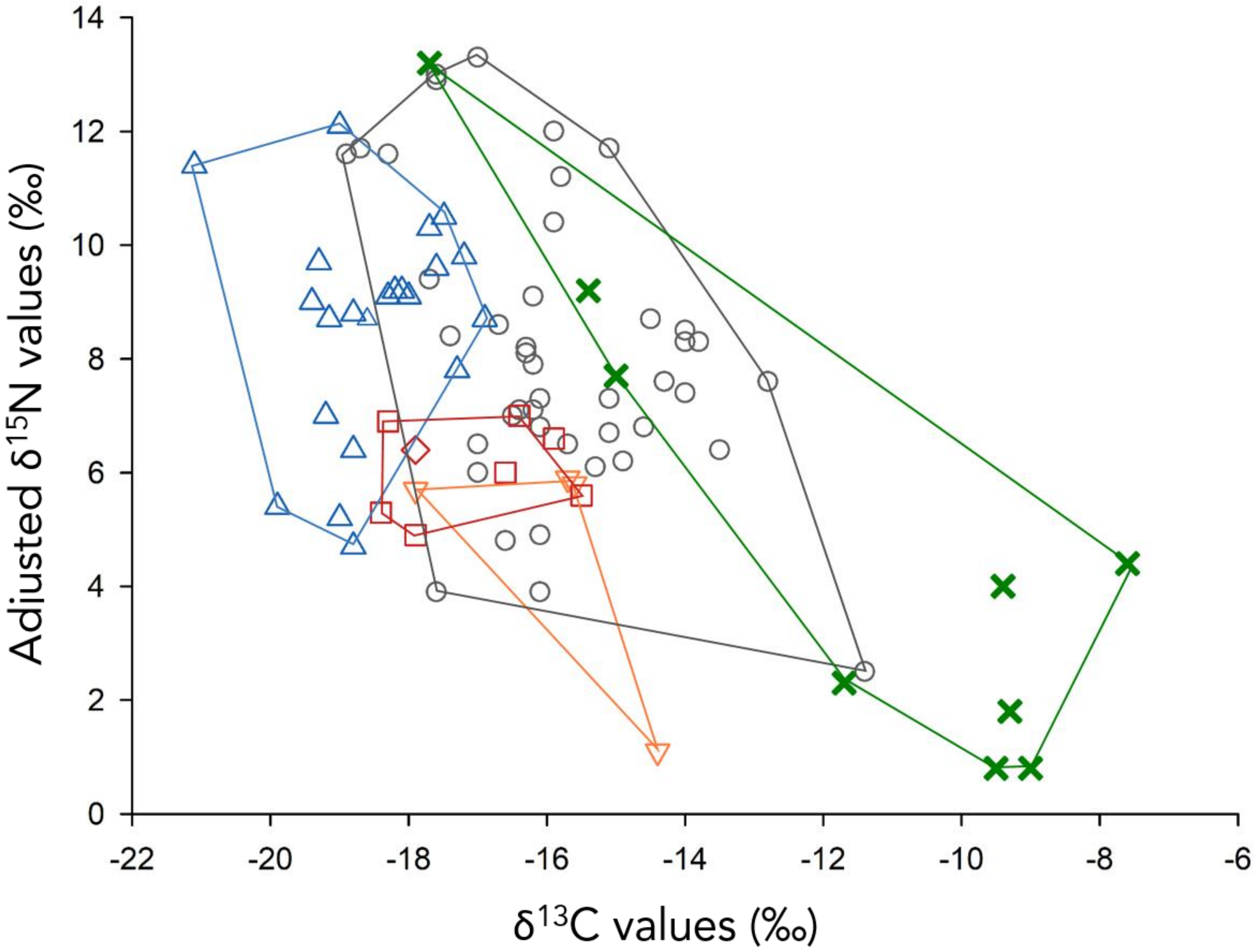

Fig. S3. Scatterplot of 91 means from values of $\delta^{13}C$ and adjusted values of $\delta^{15}N$ [adjusted using baseline phytoplankton data extracted from Pethybridge *et al.* (2018), see Table S6] in adults of six marine turtle species (*C. caretta*, dark grey circle; *C. mydas*, green cross; *D. coriacea*, blue triangle; *E. imbricata*, orange inverted triangle; *L. kempii*, red diamond, *L. olivacea*, red open square). Each point represents a single population. A maximum convex hull is drawn around all points for a given species to facilitate visual comparison. A comparison with Fig. 5 shows a similar pattern except for *L. olivacea*.
